# Organoids glimpse: the nexus for diverse tumor heterogeneity

**DOI:** 10.3389/fcell.2026.1817560

**Published:** 2026-04-28

**Authors:** Jinyang Xu, Qiushi Feng, Yangyang Xia, Lingzi Liao, Ziwei Dai, Zhigang Cai, Shang Xie

**Affiliations:** Department of Oral and Maxillofacial Surgery, Peking University School and Hospital of Stomatology and National Center for Stomatology and National Clinical Research Center for Oral Diseases and National Engineering Research Center of Oral Biomaterials and Digital Medical Devices, Beijing, China

**Keywords:** intertumoral heterogeneity, intratumoral heterogeneity, patient-derived organoids, PDO hub, temporal heterogeneity, tumor microenvironment

## Abstract

Intratumor, intertumor and temporal heterogeneity shapes capricious therapeutic response and complicates personalized treatment. It reflects interacting genetic, epi-genetic, transcriptional, and microenvironmental variation. Patient derived organoids (PDOs) are three-dimensional, patient anchored cultures that preserve key tumor programs while enabling scalable perturbations and high dimensional readouts, making them well suited for mechanistic dissection of heterogeneity. PDO panels across the intratumoral axis resolve niche linked molecular and functional states within edge core organization, perivascular territories, differentiation hierarchies, and behavioral programs including metabolism, immune evasion, invasion, and proliferative control. At the intertumoral axis, PDO panels translate interpatient and interlesional diversity into reproducible spectra of pathway dependence and drug vulnerability and enable mechanism-based stratification. In parallel, along the temporal axis, longitudinal and drug challenged organoids reconstruct treatment shaped state transitions and re-sistance trajectories on experimentally tunable time scales. The current review summarized recent evidence on PDOs, and synthesized studies that integrate organoid culture with multiomics profiling, functional perturbation, and longitudinal sampling. It delineated key boundary conditions, including incomplete microenvironmental context, workflow variability, establishment bias, and limits in mapping *ex vivo* response to clinical outcomes. On this basis, this review proposes a cooperative multi model research ecology in which PDOs serve as central molecular workhorses linking heterogeneity maps to mechanism informed therapeutic strategies.

## 1 Introduction

Tumor heterogeneity is a defining feature of cancer and is manifested as complex variation across the tumor system at the genetic, epigenetic, cellular state, and functional phenotypic levels. It’s a major source of variability in disease progression, therapeutic response, and clinical outcome (Ding et al., 2022; Dagogo-Jack and Shaw, 2018; Hendriks et al., 2024). Traditionally, this heterogeneity can be divided into interpatient heterogeneity and intrapatient heterogeneity (Meacham and Morrison, 2013; Veneziani et al., 2023; Seferbekova et al., 2023; Jacob et al., 2020). The former refers to the biological differences observed among tumors of the same histological type in different patients, whereas the latter refers to the marked differences between primary and metastatic lesions within the same patient, as well as among distinct spatial regions and cellular subpopulations within a single tumor (Meacham and Morrison, 2013; Nishimura et al., 2023). In recent years, with the deepening of research, tumor heterogeneity has increasingly been understood as a spatiotemporally dynamic evolutionary process grounded in genetic alterations, amplified by epigenetic remodeling, and continuously driven by microenvironmental selection and therapeutic pressure (Dagogo-Jack and Shaw, 2018; Nishimura et al., 2023; Laisné et al., 2025; Sibai et al., 2026; Dayton et al., 2023).

However, capturing this multidimensionality remains a formidable challenge for conventional preclinical models (Micoli et al., 2025). Conventional preclinical models, including genetically engineered mouse models (GEMMs), two-dimensional (2D) culture systems, spheroids and multicellular aggregates, *ex vivo* tissue slices, and computational models, each involve trade-offs among tumor fidelity, cost, and experimental time. In this context, patient derived organoids (PDOs) have emerged as a versatile intermediate platform, because they preserve key features of tumor architecture and lineage organization (Pagliaro et al., 2025; Bhatia et al., 2022; Wang D. et al., 2025), striking a balance between biological fidelity and experimental manipulability (Mao et al., 2024; Kluiver et al., 2024; Diao et al., 2024; Alieva et al., 2024; Nguyen et al., 2024; Lo et al., 2025; Uijtte et al., 2025; Herpers et al., 2022; Tebon et al., 2023). By integrating PDOs with advanced multi-omics and longitudinal profiling, it is now increasingly feasible to dissect the mechanisms underlying tumor heterogeneity across multiple scales (Micoli et al., 2025; Yue et al., 2025; Xu et al., 2023; Fitzpatrick et al., 2023; LeBlanc et al., 2022; Al Shihabi et al., 2024; Ren et al., 2023).

**FIGURE 1:**
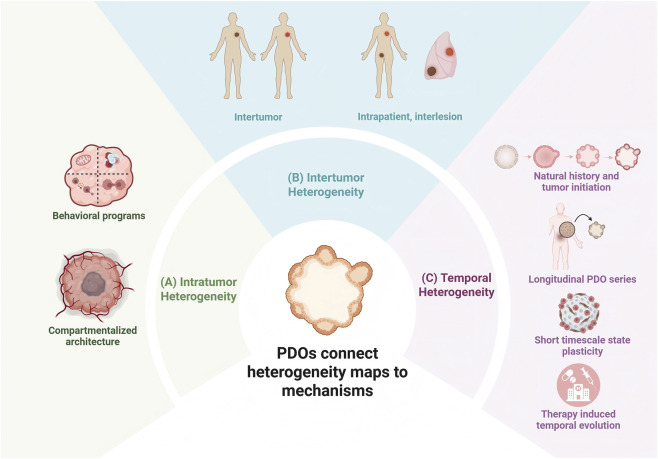
Tumor heterogeneity organized along intratumoral, intertumoral, and temporal axes and operationalized in PDO systems. **(A)** Spatially structured and program-level intratumoral states. **(B)** Cross-patient and interlesional variation. **(C)** Time-dependent remodeling during progression and treatment, including plasticity and resistance evolution. PDOs connect descriptive heterogeneity maps to testable mechanisms under controlled conditions. The image was created by BioRender.

Given the rapid technological advancements in this space, a timely synthesis of how organoids are reshaping cancer research is warranted. This review provides a narrative but mechanism oriented synthesis of how PDOs can serve as experimentally tractable models for studying tumor heterogeneity across three major dimensions, namely intratumoral, intertumoral, and temporal heterogeneity, as outlined in Figure 1. Rather than exhaustively surveying all oncologic applications of organoid systems, we focus on mechanistically informative studies in solid tumors, including colorectal cancer, pancreatic cancer, glioblastoma, and other solid tumor types. These studies combine PDOs with molecular, functional, and context aware analytical approaches to investigate tumor heterogeneity. The literature discussed here is centered mainly on studies published from 2022 to 2025, while incorporating a limited number of earlier landmark studies to provide conceptual context and methodological grounding. We also assess the current limitations of PDO based models and consider their translational relevance for patient stratification, drug development, and adaptive trial design.

## 2 Origins and manifestations of tumor heterogeneity

### 2.1 Underlying mechanisms of tumor heterogeneity

At the genomic level, the essence of tumor heterogeneity lies in the accumulation, temporal ordering, and selection of divergent somatic alterations, including driver mutations, arm level and focal copy number changes, whole genome doubling, chromosomal instability, and extrachromosomal DNA amplification. These events define the phylogenetic scaffolds along which subclones expand, compete, and seed later phenotypic divergence (Frankell et al., 2023; Leshchiner et al., 2023; McPherson et al., 2025; Wang Z. et al., 2024). Taking head and neck squamous cell carcinoma as an example, Leshchiner et al. reconstructed its early genetic progression and showed that, even in the absence of premalignant tissue, the relative timing of mutations, arm level copy number alterations, human papillomavirus integration, and transitions to aneuploidy can be inferred from bulk tumor sequencing (Leshchiner et al., 2023). Their analysis further linked transitions to aneuploidy with increased intratumor genetic heterogeneity and shorter overall survival. In parallel, Frankell et al. analyzed 1,644 tumor regions from the first 421 patients enrolled in TRACERx and showed that, in non-small cell lung cancer, many common cancer genes were under significant subclonal selection, subclonal whole genome doubling was frequent, and copy number heterogeneity was associated with early extrathoracic relapse (Frankell et al., 2023). These studies indicate that DNA based heterogeneity is not merely an end point of tumor progression, but is laid down through ordered evolutionary steps that continue to shape clinical behavior.

Beyond the mutational and early lineage defining events described above, copy number evolution also represents a critical dimension of tumor heterogeneity. Wang et al. further clarified its importance by analyzing 184 whole genome sequenced samples from 75 patients across five tumor types and found that clonal copy number gains frequently occur late, often just before the clonal expansion captured at sampling, and in more than 60 percent of cases after genome doubling (Wang Z. et al., 2024). This observation supports a model in which early initiating lesions establish the malignant lineage, whereas later copy number gains accelerate expansion and diversify the resulting clone. Convergent evidence for the evolutionary role of chromosomal instability came from renal cancer, where Perelli et al. used somatic mosaic genetically engineered models and cross species analysis to show that conserved patterns of aneuploidy drive malignant progression and that metastatic tumors converge on a chromosomal instability tolerant state through dysregulation of interferon signaling (Perelli et al., 2023). At a more mechanistic level, Cosenza et al. developed a machine learning assisted platform that coupled live cell imaging with single cell genomics and showed that dicentric chromosomes can function as initiating lesions for *de novo* chromosome alterations that accumulate over successive cell cycles (Cosenza et al., 2025). Importantly, this chromosome level diversification can be further amplified by ongoing whole genome doubling. Using single cell whole genome sequencing of 70 high grade serous ovarian cancer samples from 41 patients, representing 30,260 tumor genomes, McPherson et al. found near ubiquitous evidence that whole genome doubling can remain an ongoing mutational process (McPherson et al., 2025). Tumors with ongoing whole genome doubling displayed increased cell to cell diversity together with higher rates of chromosomal missegregation and micronucleation. In lung cancer, Pawlik et al. used ALPACA to reconstruct clone specific copy number evolution in the TRACERx 421 cohort and found increased chromosomal instability in metastasis seeding clones, with enrichment of losses affecting tumor suppressor genes and amplifications affecting CCND1 (Pawlik et al., 2025). They further showed that clone copy number diversity was linked to survival. Collectively, these studies indicate that chromosome level genetic events, including copy number evolution, chromosomal instability, and ongoing whole genome doubling, are major genomic drivers of tumor heterogeneity.

Recent work has also expanded the concept of DNA based heterogeneity beyond chromosomal alterations to include extrachromosomal DNA. Kim et al. analyzed 8,060 newly diagnosed primary cancers, untreated metastases, and heavily pretreated tumors and found that extrachromosomal DNA amplifications were significantly more frequent in untreated metastatic and pretreated tumors than in newly diagnosed cancers (Kim et al., 2024). In longitudinally matched samples, extrachromosomal DNA amplicons were more likely to be retained over time than chromosomal amplifications. At pan cancer scale, Bailey et al. examined 14,778 patients across 39 tumor types in the 100,000 Genomes Project and showed that 17.1 percent of tumor samples contained extrachromosomal DNA, underscoring that this is not a rare structural anomaly but a recurrent source of oncogene amplification and adverse outcome (Bailey et al., 2024). A particularly illustrative disease specific example came from pancreatic ductal adenocarcinoma, where Fiorini et al. demonstrated that extrachromosomal DNA is a major source of MYC heterogeneity and that variable MYC dosage allows cancer cells to adapt rapidly and reversibly to microenvironmental changes (Fiorini et al., 2025). These findings highlight that tumor heterogeneity at the DNA level is not only encoded by chromosomal mutations and copy number alterations, but can also be actively amplified through extrachromosomal elements.

Overall, current evidence supports the view that the heterogeneous steps are from DNA level change. Early driver mutations and broad copy number events define the initial evolutionary framework. Subsequent chromosomal instability, whole genome doubling, and late copy number gains expand clonal diversity and can bias metastatic selection. Extrachromosomal DNA provides an additional route to focal amplification and rapid genetic diversification. Importantly, these DNA based processes explain why tumors often contain multiple genetically distinct subclones, but they do not fully explain why related clones can still diverge in cell state, lineage identity, and therapeutic phenotype. That gap provides the rationale for considering epigenetic regulation as a second major source of heterogeneity.

### 2.2 Epigenetic drivers of tumor heterogeneity

Built upon the genetic framework that defines long term clonal architecture, epigenetic regulation serves as a complementary source of tumor heterogeneity by enabling more rapid, adaptive, and reversible changes in cellular state. Mechanisms such as altered chromatin accessibility, enhancer rewiring, DNA methylation, histone modifications, and three-dimensional genome reorganization reshape transcriptional programs and cellular identity, thereby enabling phenotypic divergence even among genetically related tumor cells. A recent pan-cancer study by Terekhanova et al. constructed an epigenetic and transcriptomic atlas across 11 tumor types using single-nucleus chromatin accessibility and matched single-cell or single-nucleus transcriptomic profiling, and identified both shared and cancer-specific epigenetic drivers associated with cancer transitions (Terekhanova et al., 2023). Notably, TP53, hypoxia, and TNF signaling were linked to cancer initiation, whereas epithelial-mesenchymal transition and apical junction programs were more closely associated with metastatic transition, underscoring that epigenetic remodeling is not merely a passive consequence of oncogenic mutations but an active determinant of tumor state diversification.

One important implication is that epigenetic heterogeneity can arise not only from intrinsic dysregulation of chromatin regulators, but also from local ecological cues. In colorectal carcinoma, Zhou et al. identified a recurrent super-enhancer activated in epithelial cancer cells by inflammatory signals in the local tumor microenvironment, rather than by a purely cell-intrinsic mechanism. This enhancer drove PDZK1IP1 expression and increased reductive capacity through the pentose phosphate pathway, thereby conferring a growth advantage under oxidative conditions. The study is particularly informative because it shows that the tumor microenvironment can directly impose an oncogenic enhancer state on cancer cells, providing a concrete mechanism by which spatially distinct microenvironments may generate epigenetically distinct tumor subpopulations (Zhou et al., 2022). Consistent with this view, Becker et al. showed in colorectal tumorigenesis that polyps and carcinomas are organized along a continuum of epigenetic and transcriptional change, with progressive expansion of stem-like states and DNA methylation changes that are strongly anti-correlated with accessibility changes across malignant progression (Becker et al., 2022). Epigenetic heterogeneity is a key mediator through which local microenvironmental differences are translated into spatially and functionally distinct tumor cell states, thereby contributing to intratumoral heterogeneity beyond what can be explained by stable genetic divergence alone.

A related layer of heterogeneity lies in the remodeling of regulatory architecture itself. In early colorectal carcinogenesis, Zhu et al. generated high-resolution chromatin conformation maps from 33 colon samples spanning unaffected mucosa, dysplastic polyps, and adenocarcinoma, and found a progressive global loss of promoter-enhancer connectivity during neoplastic progression (Zhu et al., 2024). The authors proposed a two-phase model in which neoplastic transformation shifts promoter-enhancer interactions from a redundant state toward a rate-limiting one for transcription, thereby changing how epigenetic perturbations are translated into gene expression. Extending this architectural perspective to single-cell resolution, Liu et al. traced the evolution of three-dimensional genomes in Kras-driven lung and pancreatic cancers and identified stage-specific alterations in genome compaction, compartmentalization, and heterogeneity (Liu M et al., 2025). Remarkably, single-cell 3D genome architectures were sufficient to distinguish morphologic cancer states despite substantial cell-to-cell variation, further supporting the view that higher-order chromatin organization is itself a functional layer of tumor heterogeneity. Remodeling of regulatory and three-dimensional chromatin architecture provides an additional epigenetic basis for tumor heterogeneity by enabling genetically related cells to adopt divergent transcriptional outputs and phenotypic states during tumor progression.

DNA methylation provides another major route by which epigenetic regulation cooperates with tumor evolution. In the TRACERx study, Gimeno-Valiente et al. performed reduced representation bisulfite sequencing on 217 tumor and matched normal regions from 59 patients with non-small cell lung cancer and developed an integrative framework to analyze methylation evolution alongside DNA and RNA data (Gimeno-Valiente et al., 2025). Their work directly addresses a central conceptual issue in tumor heterogeneity research, namely that methylation evolution does not proceed independently of genomic evolution but instead cooperates with it during tumor progression. This point is particularly important for the present review because it supports the view that DNA-based and epigenetic origins of heterogeneity are mechanistically intertwined rather than hierarchically separated. A further extension of this principle was provided by Lakatos et al., who analyzed 29 colorectal cancers at single-gland resolution using whole-genome sequencing, RNA sequencing, ATAC-seq, targeted sequencing, and cyclic immunofluorescence (Lakatos et al., 2025). They showed that chromatin architecture can suppress the expression of neoantigens and antigen-presenting machinery, thereby driving early immune evasion in a gland-specific manner. Thus, epigenetic heterogeneity not only shapes lineage states and transcriptional programs, but also extends directly into tumor–immune coevolution. These findings therefore support the view that epigenetic heterogeneity is a complementary and adaptive layer of heterogeneity that interacts with genetic background to diversify malignant cell states and their immune phenotypes over relatively short timescales.

Epigenetic drivers are especially evident in tumors undergoing lineage plasticity under therapeutic pressure. In treatment-emergent neuroendocrine prostate cancer, Baca et al. profiled histone modifications in patient-derived xenografts and identified approximately 15,000 candidate regulatory elements recurrently activated in neuroendocrine disease (Baca et al., 2021). They further showed that the FOXA1 cistrome is extensively reprogrammed, with loss of FOXA1 binding at prostate adenocarcinoma-enriched regulatory elements and gain of FOXA1 occupancy at neuroendocrine-enriched sites. In parallel, Cejas et al. demonstrated that neuroendocrine prostate cancer is not a uniform state but contains at least two major epigenetic subtypes, defined by ASCL1 and NEUROD1, which can coexist as genetically and epigenetically distinct subpopulations within the same human metastasis (Cejas et al., 2021). More recently, Lu et al. showed that neuroendocrine prostate cancers and castration-resistant adenocarcinomas display distinct three-dimensional chromatin architectures (Lu et al., 2025). Mechanistically, FOXA2 initiated binding at neuroendocrine enhancers, induced regional DNA demethylation, and promoted NKX2-1 expression; NKX2-1 then cooperated with FOXA2 through chromatin looping and recruitment of p300/CBP to activate neuroendocrine enhancer programs. Taken together, these studies provide a particularly clear illustration of how epigenetic remodeling can drive lineage switching, subtype diversification, and therapy-associated state transitions even when tumors remain closely related at the genomic level.

Through context-dependent enhancer activation, progressive methylation remodeling, architectural rewiring of promoter-enhancer interactions, and lineage-specific transcription factor circuits, epigenetic mechanisms help explain why tumors sharing similar genetic backgrounds can nevertheless diverge in cell state, immune phenotype, metastatic competence, and therapeutic response.

### 2.3 Phenotypic manifestations of tumor heterogeneity

Tumor heterogeneity is phenotypically manifested as spatially and functionally organized variation within tumors. These phenotypic states are not randomly distributed, but are often structured by local ecological niches and become evident through edge core organization, perivascular territories, differentiation hierarchies, and behavioral programs including metabolism, immune evasion, invasion, and proliferative control. Framing tumor heterogeneity at this phenotypic level is important because it connects underlying molecular diversification to the observable and clinically relevant behaviors of malignant tissues.

One major manifestation of intratumoral heterogeneity is edge–core organization, in which tumor cells at the core and those at the invasive front occupy distinct niche-associated molecular and functional states. In colorectal cancer, Househam et al. showed that spatially separated regions within the same tumor frequently display substantial transcriptional divergence, supporting that edge- and core-associated phenotypes can emerge as context-dependent states rather than as simple readouts of genetic lineage alone (Househam et al., 2022). The same study further found that spatial intermixing of clones was common, but that tumor growth patterns differed across cases, with some lesions expanding more uniformly and others preferentially at the periphery, indicating that the tumor edge can act as a distinct ecological compartment with different growth dynamics from the inner tumor mass. Consistent with this spatially structured view, Mo et al. analyzed tumor microregions across six cancer types and directly compared transcriptional programs between microregion centres and leading edges. They found recurrent enrichment of metabolic activity in tumor centres, whereas leading edges showed increased antigen-presentation programs and were further characterized by genes linked to migration, invasion, and immune modulation (Mo et al., 2024).

A further manifestation of intratumoral heterogeneity lies in perivascular territories, where tumor cells and surrounding stromal and immune components assemble into niche-specific molecular and functional states. In malignant gliomas, Ren et al. identified a vascular niche that was distinct from tumor core, invasive, and hypoxic regions and was enriched for pericyte/endothelial cells and microglia/macrophages, indicating that perivascular territories represent multicellular ecosystems rather than purely anatomic locations (Ren et al., 2023). Consistently, Karimi et al. showed in glioblastoma that perivascular tumor cells and macrophages display distinct proliferative and immune-regulatory features compared with cells away from vessels, supporting the existence of a functionally specialized vascular microniche (Karimi et al., 2023). Extending this concept to brain metastasis, Gan et al. demonstrated that metastatic breast cancer cells can adopt a perivascular growth architecture with distinct stromal and microglial interactions during brain colonization (Gan et al., 2024). Together, these studies indicate that perivascular territories are a specific phenotypic manifestation of tumor heterogeneity.

Phenotypic heterogeneity is also evident in differentiation hierarchies, where genetically related tumor cells occupy distinct yet interconnected lineage or cell-state positions. Such hierarchies are a major way in which tumors diversify without requiring complete genetic separation between subpopulations. In glioblastoma, Migliozzi et al. showed that tumor cell state coherence was strongest in homotypic clusters with defined relationships to neighboring non-malignant cells, whereas dispersed cells were more likely to lose their original state, acquire alternative phenotypes, and remodel their surrounding microenvironment (Migliozzi et al., 2025). This finding indicates that differentiation state is not determined solely by lineage history, but is continuously reinforced or destabilized by local niche structure. A related hierarchical pattern was reported in colorectal tumorigenesis by Zheng et al., who used single-cell transcriptomics to define physiological, benign, and malignant epithelial states and further identified adenoma-precursor and carcinoma-precursor populations along the normal–adenoma–carcinoma sequence. In hepatocellular carcinoma, Chu et al. identified three recurrent cancer cell archetypes—metabolism, stemness, and inflammation—with distinct spatial distributions and ecological interactions (Chu et al., 2025). These archetypes imply that intratumoral diversity can be organized as a hierarchy of partially interchangeable cell states rather than as a uniform malignant population. Thus, differentiation hierarchies constitute a central phenotypic manifestation of tumor heterogeneity, linking lineage plasticity to spatial ecology and local tissue context.

Beyond structural and hierarchical organization, tumor heterogeneity is further manifested through behavioral programs. These programs represent perhaps the most clinically meaningful level of phenotypic divergence, because they directly determine how tumor cells grow, survive, invade, evade immunity, and respond to therapy.

Metabolic heterogeneity is one important example. In the pan-cancer spatial analysis by Mo et al., metabolic activity was preferentially enriched in central tumor regions, indicating that metabolic programs can be spatially partitioned within individual lesions (Mo et al., 2024). In hepatocellular carcinoma, the metabolism archetype defined by Chu et al. formed an immunosuppressive niche together with TREM2-positive tumor-associated macrophages, restricting CD8 effector-memory T-cell infiltration and promoting invasion, resistance to effector cytokines, and poor immunotherapy response (Chu et al., 2025). These data indicate that metabolic programs are tightly linked to local immune suppression and invasive behavior rather than being purely cell-autonomous traits.

Immune evasion provides another major behavioral manifestation of heterogeneity. Different tumor regions may vary markedly in antigen presentation, immune-cell recruitment, cytokine responsiveness, and susceptibility to immune killing. As noted above, Mo et al. found enhanced antigen-presentation activity at tumor leading edges, whereas De Zuani et al. identified intratumoral regions enriched for anti-inflammatory macrophages and weakened NK-cell function in lung cancer (De Zuani et al., 2024). In small cell lung cancer, Chen et al. analyzed 165 tumors using CODEX and multi-omics profiling and identified a multi-positive tumor-cell neighborhood within the ASCL1-positive subtype that was characterized by high SLFN11 expression and associated with poor prognosis (Chen H. et al., 2025). In the same study, a spatially assembled immune niche composed of antitumoral macrophages, CD8 T cells, and natural killer T cells correlated with superior survival and better response to immunotherapy. These findings show that immune evasion is not a uniform tumor-wide property, but a regionally organized behavioral program embedded within heterogeneous tumor ecosystems.

Invasion is likewise heterogeneously distributed across tumors. The preferential peripheral expansion observed by Househam et al. in a subset of colorectal cancers suggests that proliferative behavior can vary across spatial compartments within a single lesion (Househam et al., 2022). At the same time, the invasive and cytokine-resistant features of the metabolism-associated hepatocellular carcinoma archetype described by Chu et al. indicate that invasive capacity is closely linked to specific ecological and transcriptional states rather than being equally shared by all malignant cells (Chu et al., 2025). Proliferation and invasion should be understood as niche-linked behavioral programs that emerge unevenly across tumor regions and contribute directly to differences in progression, dissemination, and treatment response.

Proliferative control is another important behavioral manifestation of tumor heterogeneity, as proliferative activity is not uniformly maintained throughout the malignant population but varies across clones, spatial regions, and local niches. Lucas et al. showed that proliferation rates can differ among evolutionarily distinct clones coexisting within the same tumor, indicating that proliferative behavior is itself a clonal variable (Lucas et al., 2025). At the spatial level, Wang et al. found that triple-negative breast cancers contain highly proliferative large tumor patches in specific molecular subtypes, suggesting that proliferative activity can be regionally concentrated within individual lesions (Wang et al., 2024a). Karimi et al. showed in glioblastoma that perivascular tumor cells display altered Ki67:CC3 ratios compared with tumor cells away from vessels, indicating that local vascular territories can further modulate the balance between proliferation and cell loss (Karimi et al., 2023). Proliferative control is a structured phenotypic manifestation of tumor heterogeneity, shaped jointly by clonal background, spatial organization, and microenvironmental context.

Taken together, current evidence suggests that the phenotypic manifestations of tumor heterogeneity are best understood as niche-linked molecular and functional states distributed across edge–core organization, perivascular and stromal territories, differentiation hierarchies, and behavioral programs including metabolism, immune evasion, invasion, and proliferative control. This perspective is important because it moves the discussion of heterogeneity beyond static molecular differences and toward the spatially organized phenotypes through which tumors actually grow, adapt, metastasize, and resist therapy.

### 2.4 Preclinical models in tumor heterogeneity development

Before the widespread adoption of PDOs, tumor heterogeneity was investigated through a range of preclinical models. Ideally, such models should preserve key histological and molecular features of the tumor of origin, remain sufficiently scalable for laboratory use, and provide reliable prediction of therapeutic response. In practice, however, no single model fully satisfies all of these requirements (Durinikova et al., 2021). For this reason, understanding how these earlier models contributed to heterogeneity research is essential for clarifying both the foundations and the remaining gaps that later motivated the development of PDO-based platforms.

#### 2.4.1 *In vivo* models: induced tumors, GEMMs, xenografts, and syngeneic systems

Before patient-derived organoids became widely used, *in vivo* models provided the earliest experimental settings in which tumor heterogeneity could be followed under physiological selection. Among these, induced tumor models and genetically engineered mouse models were particularly important for linking driver events to stepwise tumor evolution. For example, CRISPR-mediated *in situ* liver cancer modeling in non-human primates demonstrated that complex primary and metastatic lesions can be generated directly in the native tissue environment, thereby preserving the evolutionary pressures imposed by vasculature, immunity, and organ-specific stromal context (Zhong et al., 2021). More recently, Müller et al. established a suite of genetically driven immunocompetent liver cancer models and showed that these systems recapitulate clinically relevant features of human hepatocellular carcinoma, including clonal origin, histopathological diversity, and metastatic behavior, while cross-species transcriptomic integration resolved four conserved human–mouse subtype clusters (Müller et al., 2025). The major strength of inducible and genetically engineered models in heterogeneity research is that they allow tumor diversification to be studied as a process unfolding under intact tissue architecture and systemic selection, rather than as a phenotype measured only after excision. Their limitation, however, is equally clear. Because the initiating lesions are experimentally predefined, these models can oversimplify the mutational starting point of human disease and may not fully capture the breadth of patient-specific genomic contingencies.

Xenograft models addressed a different need, namely the preservation of human tumor material *in vivo*. In multi-region non-small cell lung cancer, Hynds et al. established 48 patient-derived xenografts from 22 TRACERx patients and showed that most grafts retained the histological appearance of the source tumor, but model establishment imposed a genomic bottleneck such that individual grafts often represented only a single tumor subclone (Hynds et al., 2024). Importantly, different subclones could still be recovered by grafting different tumor regions, which means that PDX models can preserve clinically relevant recent evolutionary history, but only if heterogeneity is sampled prospectively rather than assumed to be contained in a single fragment. This is precisely why PDXs remain valuable in heterogeneity studies but are insufficient as one-to-one surrogates of the whole tumor. They preserve human architecture and selective pressures better than 2D systems, yet they incompletely represent full intratumoral diversity, are slow and costly to establish, and replace much of the human stromal and immune context with murine counterparts over time (Migliore et al., 2025).

Syngeneic tumor models contribute another distinct layer by retaining an intact immune system. In high-grade serous ovarian cancer, Elorbany et al. used syngeneic models together with single-cell profiling to show that anti-STAB1 and FOXP3 antisense strategies reshaped the tumor microenvironment, increased survival, and produced durable resistance to tumor rechallenge (Elorbany et al., 2024). The value of such models for heterogeneity research is not that they reproduce patient-specific clones, but that they reveal how heterogeneous immune niches are generated, maintained, and therapeutically remodeled when host immunity remains functional. Their major shortcoming is that the tumor cells are murine rather than human, so the resulting heterogeneity is biologically informative for immune ecology and response dynamics, but less suitable for directly extrapolating patient-specific genotype–phenotype relationships.

#### 2.4.2 *In vitro* models: tumor cell lines, multicellular spheroids, scaffold systems, and tumor-on-a-chip

*In vitro* systems were developed to complement *in vivo* models by increasing experimental controllability. Classic tumor cell lines and 2D culture models are often criticized for oversimplification, but they remain useful for dissecting state transitions and non-genetic heterogeneity under tightly controlled perturbation. A representative example is provided by Dai et al., who developed ReSisTrace in ovarian cancer cells and showed that treatment-primed resistant states can be detected before exposure to olaparib, carboplatin, or natural-killer-cell-mediated killing by exploiting transcriptomic similarity between sister cells (Dai et al., 2024). Likewise, Contreras-Trujillo et al. integrated lineage barcoding, single-cell profiling, and functional assays to connect heterogeneous transcriptional states with growth, metastasis, and treatment response (Contreras-Trujillo et al., 2021). These studies show why 2D systems remain experimentally powerful: they are uniquely suited for high-resolution perturbation and causal dissection of non-genetic heterogeneity. Their limitation is that they poorly represent three-dimensional architecture, cell–matrix signaling, and multicellular ecological constraints, so the heterogeneity they reveal is often real but incomplete.

Three-dimensional spheroids and multicellular tumor spheroids were introduced to partially restore these missing constraints. In a high-resolution microfluidic assay using individual cancer spheroids, Ronteix et al. showed that the first recruited T cells triggered a positive-feedback loop that accelerated later recruitment and that tumor killing depended on cooperative rather than purely additive immune behavior (Rontei et al., 2022). This is a useful example because it shows that spheroid systems can reveal heterogeneous collective behaviors that monolayer models cannot capture. However, the biological interpretation of such models must remain cautious. Spheroids restore gradients and cell–cell interactions, but they still simplify tissue composition and rarely preserve the stromal or immune diversity observed in patient lesions (Commander et al., 2020; Konen et al., 2017). They are therefore best regarded as intermediate systems for studying emergent group behavior rather than faithful reconstructions of patient-specific tumor ecology.

Scaffold-based systems and tumor-on-a-chip platforms extend this logic by imposing more realistic mechanical and spatial constraints. In glioblastoma, Neufeld et al. built a perfusable 3D-bioprinted scaffold containing tumor cells together with astrocytes and microglia and showed that the resulting constructs reproduced proliferation kinetics, therapy responses, and molecular features closer to orthotopic xenografts than simpler cultures (Neufeld et al., 2021). In parallel, Perucca et al. developed the fully humanized MIRO platform, in which cancer spheroids, fibroblasts, extracellular matrix, and immune cells were spatially organized to model the tumor–stroma interface (Perucca et al., 2025). Their work demonstrated that stromal barriers actively guide immune exclusion and that interleukin-2 can partially restore trastuzumab-induced cytotoxicity in resistant immune-excluded settings. These models are particularly informative for heterogeneity research because they allow investigators to manipulate physical constraints and stromal composition in a modular way, thereby testing how niche geometry and cell positioning shape divergent phenotypes. Their central drawback is complexity without full tissue fidelity: although they outperform simple culture systems in spatial control, they remain engineered assemblies whose stromal ratios, matrix properties, and flow conditions are still experimentally chosen rather than naturally evolved.

#### 2.4.3 *Ex vivo* models: tumor slice culture and patient-derived explants

*Ex vivo* systems occupy an important middle ground because they preserve native tumor architecture and multicellular context while remaining experimentally accessible. Among these, tumor slice cultures and patient-derived tumor fragments are particularly informative for heterogeneity research because they retain pre-existing spatial relationships among malignant cells, stromal cells, vasculature, and immune populations rather than reconstructing them after tissue dissociation. In human colorectal cancer liver metastasis slice cultures, Sullivan et al. showed that interleukin-10 blockade produced a 1.8-fold increase in T-cell-mediated carcinoma cell death, and that this effect depended on intact MHC-I and MHC-II antigen presentation, directly illustrating how slice cultures can uncover clinically relevant variation in immune susceptibility within preserved metastatic niches (Sullivan et al., 2023). In primary liver cancer, Jagatia et al. used patient-derived precision-cut tissue slices to maintain tissue architecture, tumor viability, and an immunocompetent microenvironment for longitudinal assessment over 8 days of *ex vivo* culture, enabling patient-specific evaluation of both single-agent and combination treatment responses (Jagatia et al., 2023). A complementary *ex vivo* fragment approach was reported by Voabil et al., who developed a patient-derived tumor fragment platform to dissect the early immunological response of human tumor tissue to PD-1 blockade, showing that preserved tumor ecosystems can reveal whether intratumoral immune cells remain functionally reactivatable (Voabil et al., 2021). These studies show that *ex vivo* models are especially useful for interrogating pre-existing regional, stromal, and immune heterogeneity under short-term treatment conditions. Their major limitation, however, is that they are short-lived and relatively low-throughput, making them better suited for testing existing heterogeneity than for modeling the long-term emergence of clonal diversification and adaptive evolution.

#### 2.4.4 Computational and *in silico* models

Computational and *in silico* frameworks add a different but complementary dimension to tumor heterogeneity research. Rather than preserving living tissue architecture directly, these approaches integrate multi-layered datasets to reconstruct hidden clonal structure, spatial organization, evolutionary relationships, and therapeutic vulnerabilities. Shafighi et al. developed Tumoroscope to infer clone proportions and spatial localization at close to single-cell resolution by integrating pathological images, whole-exome sequencing, and spatial transcriptomics, thereby enabling quantitative reconstruction of spatial clonal organization (Shafighi et al., 2024). Ma et al. further developed CalicoST, which infers allele-specific copy number aberrations and reconstructs tumor phylogeography from spatially resolved transcriptomic data, extending computational analysis from clonal localization to spatial evolutionary reconstruction (Ma et al., 2024). At the therapeutic level, Ianevski et al. proposed scTherapy, which uses single-cell transcriptomic profiles to prioritize multi-targeting treatment strategies across heterogeneous tumor cell populations (Ianevski et al., 2024). These models substantially improve the analytical depth of heterogeneity research by enabling spatial inference, evolutionary reconstruction, and systematic prediction of treatment response. However, they also have clear limitations. Because they are inference-based frameworks rather than living experimental systems, their performance depends heavily on the quality, resolution, and compatibility of input datasets, as well as on model assumptions and training strategies. In addition, although these approaches can generate valuable hypotheses about clonal architecture or therapeutic vulnerability, they cannot by themselves recapitulate multicellular dynamics, microenvironmental feedback, or long-term adaptive evolution, and their predictions still require validation in biological models.

Collectively, these early experimental systems established the conceptual and technical foundation for studying tumor heterogeneity, yet none offered a single experimentally accessible platform capable of preserving patient-derived architecture, supporting longitudinal manipulation, and remaining compatible with mechanistic testing. This unresolved gap helps explain why patient-derived organoids later emerged as attractive intermediate platforms for tumor heterogeneity research.

## 3 Intratumor heterogeneity revealed by tumor organoids

Intratumor heterogeneity is commonly defined as the nonuniform distribution of genetically diverse tumor subpopulations within the same lesion or across spatial regions of a tumor mass (Zeng and Jin, 2022). It can be operationalized in organoid systems at two levels. Anatomical and histopathological compartments provide a spatial coordinate system that can be translated into comparable organoid lineages, whereas behavioral programs describe how tumor cells execute niche adapted states within these compartments.

### 3.1 Anatomical and histopathological compartmentalization

From an anatomical and histopathological perspective, intratumor heterogeneity is first manifested as a set of clinically discernible spatial compartments. These spatial architectures are not only the most immediately apparent differences that pathologists observe on tissue sections, but also frequently correspond to distinct selective pressures, resource accessibility, and microenvironmental cues (Jing et al., 2025), thereby driving the emergence of stable spatially organized tumor subpopulations (Lee et al., 2025). Common spatial dimensions include: (i) the invasive tumor edge versus the tumor core, (ii)a contrast between perivascular niches and poorly perfused regions characterized by limited nutrient and oxygen supply, and (iii) well differentiated glandular structures versus poorly differentiated or stemlike areas. These anatomical dimensions are tightly coupled with genotype, epigenetic state, metabolic pathways, and the immune ecosystem, and they provide a central entry point for understanding tumor evolution (Seferbekova et al., 2023; Zeng and Jin, 2022; Kim et al., 2025).

#### 3.1.1 Edge-core differences

Tumors are not homogeneous cell masses. Rather, they resemble a pathological organ assembled from multiple functional compartments. In most solid tumors, one can readily distinguish a tumor core with higher cellular density and more severe necrosis and hypoxia, as well as an invasive front that infiltrates the surrounding tissue in finger like or sheet like patterns, also referred to as the leading edge (Fu et al., 2021). The core is typically enriched for cells under stress, metabolic rewiring, and DNA damage response programs. By contrast, the invasive front concentrates EMT and migration associated cell states, stemlike populations, extracellular matrix remodeling, and signaling linked to neural and immune interactions (Ren et al., 2023; Shard et al., 2025).

This edge core spatial axis can be naturally reconstituted at the organoid scale. As organoids increase in size, they spontaneously develop a layered organization with a proliferative outer rim and a centrally hypoxic core (Licata et al., 2023; Song et al., 2024; Hubert et al., 2016). Recent work using glioblastoma three dimensional spheroids and organoids has shown that the outer rim is enriched for highly proliferative, therapy tolerant stem like cells marked by Ki67^+^ and SOX2^+^, together with epigenetic regulators such as WDR5, whereas the inner core is enriched for HIF 1α driven hypoxia transcriptional programs and stress survival signaling (Jacob et al., 2020). This configuration recapitulates spatial metabolic and transcriptional gradients reminiscent of primary tumors (Han et al., 2024).

A multiregion sampling strategy coupled with PDO directly converts clinically annotated spatial information into a set of comparable organoid lineages. Early large-scale gastric cancer organoids have already suggested that moderately differentiated tumors often yield organoids with well-formed gland like architecture, whereas poorly differentiated or diffuse type tumors tend to form solid, more invasive spheroids. To some extent, these patterns mirror the morphological and molecular differences between well and poorly differentiated regions *in situ* (Yan et al., 2018). More recent studies have further demonstrated that an individualized tumor organoid panel built from a single patient can preserve the genetic and phenotypic diversity across spatial subregions, providing a practical framework for systematically comparing drug response profiles between core derived and invasive front derived organoids (Peng et al., 2025).

Jamaluddin et al. applied spatial proteomics across 63 sites in endometrial cancers and revealed pronounced site-specific molecular heterogeneity. They subsequently established PDOs from these distinct regions and observed that the systematic differences in morphology, growth rate, and drug sensitivity were maintained in culture. Notably, these phenotypic differences correlated strongly with the protein expression patterns of their matching primary tumor regions (Jamaluddin et al., 2022). This confirms that edge-core molecular states are heritable and can be converted into measurable phenotypes within PDOs, allowing *in situ* spatial multiomics to be functionally validated *in vitro*.

From an immunological perspective, the tumor core and the invasive front likewise exhibit a highly spatially heterogeneous immune ecosystem. Spatial transcriptomics and multiplex imaging studies have demonstrated that distinct anatomical compartments are often enriched for markedly different immune cell subsets and effector programs (Pelka et al., 2021; Oliveira et al., 2025). Organoid immune co-culture systems further allow these spatial differences to be reconstructed and perturbed under controlled conditions, and their causal effects on drug response and immune evasion to be read out directly.

In pancreatic cancer, Zhang et al. integrated single cell and spatial transcriptomics with patient derived organoid models and showed that ZEB1 recruits HDAC1 to the CXCL16 promoter, reduces promoter associated histone acetylation, and transcriptionally represses CXCL16, thereby weakening the CXCL16-CXCR6 chemotactic axis. This program is associated with an immunosuppressive tumor core marked by CD8^+^ T cell exclusion and neutrophil rich infiltration. Upon ZEB1 inhibition, GZMA^+^ CD8^+^ T cells re infiltrated the core region, and models became more responsive to gemcitabine and PD 1 blockade, with improved efficacy of CAR T based treatment in PDO and PDO derived xenograft mice settings (Zhang et al., 2025). Consistently, Lesch et al. introduced CXCR6 expressing T cells into CXCL16 high pancreatic cancer PDO and PDX models and demonstrated that the CXCL16 CXCR6 axis substantially enhances the directed migration and cytotoxicity of tumor reactive T cells toward tumor compartments, including within organoids themselves. It suggests that the same chemotactic pathway can both contribute to a cold core and be engineered to reprogram immune infiltration patterns (Lesch et al., 2021).

Organoid studies using optical metabolic imaging, provide high spatial resolution evidence for edge-core divergence at the metabolic level. Sharick et al. first showed in a mouse mammary tumor model that the optical redox ratio, computed from the intrinsic fluorescence of NAD(P)H and FAD, captures intratumoral metabolic heterogeneity at single cell resolution *in vivo*, and that organoids derived from the same tumors can recapitulate a similar spatial pattern of metabolic hot and cold spots (Sharick et al., 2019). Building on this, Gillette et al. introduced a leading-edge analysis in colorectal cancer PDO and found that FOLFOX induced shifts in the redox ratio were substantially larger within the leading-edge band than in the organoid as a whole or in the core region. These data suggest that metabolic rewiring is more pronounced at the invasive front than in the inner core, and that edge focused molecular imaging readouts can more sensitively detect treatment responses and spatial heterogeneity (Gillette et al., 2025).

Neural tumor crosstalk provides an example of how spatial analysis can be bridged to organoid based functional validation. By integrating weighted gene co-expression network analysis with single cell and spatial transcriptomics, Shard et al. identified an ion channel and γ-Aminobutyric-acid type A receptor (GABA_A_R) related module enriched at the invasive front of high-grade gliomas, and confirmed the expression of these receptor subunits in patient-derived glioblastoma explant organoids (GBOs). In GBOs, α5 selective antagonism with S44819 suppressed peripheral invasion, whereas broader partial antagonism inhibited both proliferation and invasion, with further suppression of leading-edge invasion upon combination with chemotherapy and radiation. Together, these findings suggest that a leading edge enriched GABA_A_R network is both a spatial marker of neuro synaptic heterogeneity and a druggable, front specific target in organoid models (Shard et al., 2025).

Collectively, PDOs, translate pathologist annotated edge and core compartments into parallelizable 3D experimental units. They can partially preserve region specific molecular states while enabling causal perturbations, thereby advancing spatial differences from descriptive observations to quantifiable and testable functional phenotypes.

#### 3.1.2 Perivascular versus avascular territories

Tumor angiogenesis, the formation of intratumoral vasculature through sprouting and growth of tumor associated vessels driven by dysregulated angiogenic signaling, is a long recognized yet still incompletely understood hallmark of cancer (Fares et al., 2020). Within a single tumor, perivascular regions and areas distant from vessels or devoid of vessels often constitute two sharply distinct ecological niches. Perivascular zones are typically enriched for endothelial cells, pericytes, immune cells, and extracellular matrix components, with relatively ample oxygen and nutrient supply, thereby sustaining a trophic niche that supports stemlike and highly proliferative states (Dagogo-Jack and Shaw, 2018; Onubogu et al., 2024). In contrast, regions distal to the vasculature or avascular areas experience chronic hypoperfusion, severe hypoxia, and acidosis, which more readily accumulate DNA damage and metabolic stress signals and favor quiescent or stress adapted cellular states (Seferbekova et al., 2023; Chen et al., 2023).

Recent studies combining organoids with spatial omics have clearly delineated such cellular state stratification along a perivascular to avascular axis. Greenwald et al. integrated CODEX imaging with spatial transcriptomics and single cell analyses across glioblastoma specimens and described a multilayer organization within structured regions dominated by a hypoxia gradient. The layers span a deep hypoxic compartment enriched for HIF associated programs and necrosis signatures, a hypoxia adjacent zone with inflammatory myeloid infiltration, a perivascular band enriched for vascular and myeloid immune programs, and more peripheral layers dominated by malignant lineage like states that transition toward infiltrated brain and the leading edge (Greenwald et al., 2024). In recurrent glioblastoma, Onubogu et al. combined multimodal imaging with single cell spatial transcriptomics and identified collagen rimmed vessels surrounded by immunosuppressive macrophage rich bands adjacent to fibroblast like ECM compartments, supporting an organized perivascular niche architecture (Onubogu et al., 2024). In a spatial multiomics study, Ravi further showed that distinct lineage like states in glioblastoma, such as OPC-like and MES-like programs, are strongly spatially segregated along a vascular to necrotic axis, and that this stratification is accompanied by state specific metabolic wiring and immune interaction networks (Ravi et al., 2022).

At the functional level, 3D tumor spheroids and organoid models often spontaneously develop pronounced oxygen gradients, with peripheral cells remaining relatively oxygenated while the center becomes a severely hypoxic core resembling an avascular region. Using colorectal cancer spheroids and PDO, Han et al. showed that MO 2097, a small molecule targeting hnRNPA2B1 and suppressing HIF-1α translation, exerts a stronger anticancer effect in the hypoxic zone of 3D spheroids and induces apoptosis in this region. In patient derived colon cancer organoids, responses varied across lines, indicating clinically relevant heterogeneity in sensitivity to HIF pathway targeting (Han et al., 2024).

In pancreatic cancer, Schwörer et al. showed that hypoxia potentiates an inflammatory CAF program. *In vivo*, inflammatory CAF features were enriched in pimonidazole marked hypoxic regions, whereas αSMA^+^ myCAFs were largely excluded from these areas. In KPC organoid and stellate cell coculture, genetic stabilization of HIF signaling was sufficient to shift fibroblasts toward an iCAF like state, supporting a causal role for hypoxia and HIF activity in CAF polarization (Schwörer et al., 2023).

More refined work has directly linked the perivascular niche to stemness and drug-resistant phenotypes. In ETMR, de Faria et al. used cross human-mouse single cell and spatial transcriptomics to show that RG-like and NProg-like malignant cells preferentially localize to PDGFRβ^+^ pericyte rich, ECM enriched perivascular regions, whereas more differentiated neuron like states are found farther from vessels. In a 3D forebrain organoid coculture, tumor cells could induce pericyte like differentiation and thereby help establish a perivascular niche that supports stemness and chemoresistance, which was attenuated by PDGFR inhibition *in vitro* and *in vivo* (De Faria et al., 2025).

When interrogating the causal roles of the perivascular-avascular axis, conventional PDOs lack perfused vasculature and therefore often model only one end of the spectrum, namely the avascular-hypoxic core. Here, perivascular biology can be partially approximated by diffusion-based gradients and stromal coculture, but faithful modeling of perfused niches requires vascularized or chip-based systems. Next-generation vascularized organoid platforms are emerging as a key breakthrough for dissecting perivascular biology.

Quintard et al. developed a microfluidic, multichannel vascularized organoid on chip system that stably maintains a perfused microvascular network around organoids, providing a high temporal resolution tool to study vessel guided migration (Quintard et al., 2024). Extending this concept, Du et al. introduced a personalized vascularized PDO-on-a-chip platform that integrates patient specific tumor organoids with perfusable vasculature in a single device, enabling concurrent readouts of tumor induced vascular sprouting, directed migration of tumor cells along vessels, and the dependence of these behaviors on VEGFR2-Notch signaling. They reported that highly metastatic PDOs strongly induced neovessel branching and exhibited pronounced vessel associated migratory dynamics, whereas a VEGFR2 inhibitor selectively blocked these processes. This phenotype closely matched patients’ metastatic risk, positioning the platform among the most clinically relevant perivascular niche models currently available (Du Y. et al., 2025). Notably, the vasculature itself is highly plastic in the tumor context. Guelfi et al. generated mouse vascular organoids that display an endothelial-pericyte hierarchy and perfusion capacity and undergo dynamic remodeling in response to tumor secreted cues such as VEGF and ANGPTL4, underscoring that perivascular structures are not a static backdrop but are continuously reshaped by tumor derived signals (Guelfi et al., 2025). Han et al. developed three dimensional bio-printed PDO arrays that allow systematic control of ECM, oxygen tension, and angiogenic factor environments, enabling high throughput dissection of plasticity and drug vulnerabilities along the perivascular-avascular spectrum (Han et al., 2025).

From this perspective, vascularized and gradient controlled organoid systems render the perivascular to avascular axis experimentally tractable and clinically interpretable.

#### 3.1.3 Differentiation hierarchies and lineage plasticity

Within a single tumor, cell states of different differentiation degrees often coexist and dynamically interconvert, spanning a continuum from highly differentiated, epithelial like cells that more closely resemble the tissue of origin and maintain glandular polarity and secretory functions, to poorly differentiated or dedifferentiated regions dominated by stem like and EMT or mesenchymal like programs. Recent reviews on lineage plasticity have formally positioned this differentiation-dedifferentiation spectrum as a candidate “new hallmark of cancer,” emphasizing that tumor cells can shuttle among multiple stable or quasi stable developmental related states and reprogram lineage identity to evade immune surveillance and therapeutic pressure. Mehta and Stanger argue that lineage plasticity is a central mechanism driving intratumoral heterogeneity and treatment failure, particularly evident in tumors that undergo lineage switching following targeted or hormone therapies (Mehta and Stanger, 2024). Gargiulo and Marine, from a driver passenger trailer cell state framework, redefine tumor cell states by highlighting division of labor across proliferation, invasion, and resistance, and view plasticity as the organizing logic that enables transitions among these states (Gargiulo et al., 2024). The developmental constraint model further proposes that cell states observed across many cancers are often confined to a subspace near the developmental trajectory of the cell of origin, making differentiation hierarchy a natural coordinate system for interpreting intratumoral molecular heterogeneity (Patel and Yanai, 2024). Within this framework, highly differentiated populations tend to be more sensitive to conventional cytotoxic agents and some targeted therapies, whereas poorly differentiated, stem like, and EMT-high populations exhibit greater migratory capacity, immune evasion, and drug resistance.

PDOs can preserve both cancer stem cell or stem like populations and highly differentiated epithelial cells within a shared genetic background, effectively flattening differentiation hierarchy that is otherwise entangled with tissue architecture. When combined with single cell transcriptomics, lineage tracing, and live cell imaging, PDOs enable reconstruction of intratumoral differentiation hierarchies at molecular resolution (Cheng et al., 2022). The inherent plasticity and experimental accessibility of PDOs make them an ideal substrate for single-cell transcriptomics, lineage tracing, and live-cell imaging, facilitating the reconstruction of intratumoral differentiation hierarchies at molecular resolution.

In colorectal cancer, organoid models have clearly disentangled the functional and molecular stratification between stem like or poorly differentiated states and highly differentiated epithelial cells. Cheng et al. isolated LGR4^+^CD44^+^PrPc^+^ cancer cells and showed that this subset exhibits strong tumor initiating and liver metastatic potential in orthotopic models. Single LGR4^+^CD44^+^PrPc^+^ cells could form tumor organoids under relatively low exogenous Wnt input, and derived PDOs maintained this stem like compartment while generating differentiated glandular cells expressing villin, cytokeratin 20, and mucin 2, consistent with a differentiation hierarchy within a shared genetic background. Functionally, shRNA knockdown of LGR4 or PrPc suppressed Wnt/β-Catenin signaling, reduced CD44 and Ki67, and impaired organoid formation and tumor growth, while high LGR4 expression in clinical samples associated with advanced stage and grade (Cheng et al., 2022). Concordantly, a recent large scale review of colorectal cancer CSCs by Chu et al. summarized CSC surface markers and downstream signaling axes and highlighted that the CD44^+^PrPc^+^LGR4^+^ subset plays a key role in activating Wnt/β-Catenin signaling and conferring migratory and drug resistant phenotypes (Chu et al., 2024). In addition, long term culture of mismatch repair deficient intestinal organoids with stepwise withdrawal of Wnt and EGF and BMP inhibition has been used to replay the adenoma to carcinoma sequence, suggesting that differentiation and dedifferentiation states can be rebalanced by acquired mutations and microenvironmental shifts in organoid settings, thereby supporting coupling between differentiation hierarchy and clonal evolution (Mizutani et al., 2024).

In pancreatic ductal adenocarcinoma, Papargyriou et al. cultured branched pancreatic ductal adenocarcinomas (PDAC) organoids in a collagen I matrix and obtained stable morphological families, including smooth epithelial spheroids, fine branched structures with low EMT, and highly invasive phenotypes with high EMT scores and thick branching, sometimes adopting tree like or star like architectures (Papargyriou et al., 2024). Single cell RNA sequencing and bulk transcriptomics indicated that these morphologies are not dictated by differences in driver mutations, but instead reflect a continuous state spectrum shaped by EMT intensity, adhesion and migration programs, stress responses, and metabolic rewiring. High EMT phenotypes, including thick branched, tree like, and star like organoids, showed greater metastatic potential *in vivo*, increased resistance to FOLFIRINOX, and heightened radiosensitivity, whereas epithelial like phenotypes displayed the opposite therapeutic response pattern. Together, this model illustrates across structure, transcriptome, and function that differentiation and EMT states themselves constitute a major axis shaping intratumoral molecular heterogeneity and treatment vulnerabilities in PDAC. In parallel, Bar-Hai et al. induced partial EMT with TGF-β in breast and gastrointestinal PDOs and demonstrated that organoid level cell states can be reversibly shifted along an epithelial like-partial EMT-esenchymal like continuum, thereby altering sensitivity to distinct treatment modalities (Bar-Hai et al., 2024).

Using pleomorphic adenoma of the parotid gland as an example, Xu et al. built a single cell transcriptomic atlas of pleomorphic adenoma specific epithelial cells and delineated six subpopulations spanning EMT high hybrid or stromal like clusters, a highly proliferative cluster, an EMT low MUCL1^+^ differentiated ductal epithelial cluster, and a progenitor cluster with myoepithelial, vascular, and stemness associated features. They further showed in patient derived pleomorphic adenoma organoids that the PI3K-AKT pathway is activated in the C4 CD36^+^ progenitor population, and that protein kinase B (AKT) inhibition markedly suppresses tumor sphere formation and organoid growth, indicating a targetable vulnerability of a highly plastic, poorly differentiated progenitor state (Xu et al., 2023). This work therefore connects single cell resolved differentiation hierarchies, ranging from differentiated ductal epithelium through undifferentiated progenitors to mesenchymal like states, with their functional coupling within a single tumor context. An organoid biobank for salivary gland tumors further highlighted subtype specific positioning along this ductal-progenitor-mesenchymal like continuum and demonstrated that salivary gland tumor PDOs can support genotype guided drug screening and identification of subtype specific vulnerabilities, providing evidence for bringing differentiation hierarchy concepts into precision treatment of rare head and neck tumors (Wang et al., 2022).

In aggregate, PDOs enable differentiation hierarchies and lineage plasticity to be reconstructed and perturbed within a patient anchored genetic background (Peng et al., 2025).

### 3.2 Behavioral axis based intratumoral heterogeneity

The interior of a single tumor can often be further delineated into a set of highly intertwined yet partially separable “behavioral axes”, including metabolic rewiring, immune evasion, migration/invasion programs, and proliferation dynamics (Mehta and Stanger, 2024; Pavlova and Thompson, 2016). This type of heterogeneity poses a particularly formidable challenge to conventional detection methods, as it does not always correspond to genetic divergence (Qu et al., 2024). Recent studies on eco-evolution and cellular states have further indicated that these behavioral modules exhibit a highly plastic and dynamic heterogeneous distribution within the same tumor, which is manifested in the form of cellular states, lineage plasticity, and spatial niches (Shard et al., 2025; Bareham et al., 2024; Nguyen et al., 2025).

#### 3.2.1 Metabolic rewiring

Across behavioral axes, metabolism is arguably a shared gate through which nearly every branch of intratumoral heterogeneity must pass. Classic work has distilled a diverse set of hallmarks of cancer metabolism (Pavlova and Thompson, 2016; Faubert et al., 2020). Yet these hallmarks are not a single program that is uniformly switched on or off across an entire tumor. Rather, they resemble a modular metabolic repertoire that different clones and cell states deploy in distinct combinations. At the tissue scale, these metabolic states are further finely coupled to hypoxia gradients, vascular distribution, and extracellular matrix composition. Over time, repeated pressures imposed by chemotherapy, targeted agents, or immunotherapy continuously reshuffle this metabolic mosaic, making metabolic heterogeneity a common conduit that links resistance, metastasis, and immune evasion.

Multi region isotope tracing in human lung cancer suggests that such metabolic modularity can be particularly prominent within a single tumor. Hensley et al. found marked regional differences in the utilization of glucose and lactate: some areas primarily use glucose to fuel the TCA cycle, whereas others rely heavily on circulating lactate (Hensley et al., 2016).

In a large PDAC PDO cohort, Li et al. stratified organoids into “glucomet” (glucose-dominant) and “lipomet” (lipid-dominant) subtypes. The glucomet subtype, characterized by a GLUT1/aldolase B (ALDOB)/glucose-6-phosphate dehydrogenase (G6PD) axis, exhibited chemotherapy tolerance via expanded nucleotide pools. Importantly, inhibiting GLUT1 or G6PD restored gemcitabine sensitivity, demonstrating how metabolic subtypes create distinct therapeutic vulnerabilities (Li et al., 2023).

A conceptually aligned observation emerged from Mertens et al., who conducted a drug repurposing screen in KRAS mutant colorectal cancer PDOs and showed that residual clones after standard therapy expose newly acquired dependencies on mitochondrial and one carbon metabolism, creating selective vulnerabilities to metabolic inhibitors (Mertens et al., 2023). In a more complex metastatic setting, Duan et al. used paired primary lung cancer-brain metastasis PDOs to show that the mitochondrial Hsp90 targeting Oxidative Phosphorylation (OXPHOS) inhibitor gamitrinib synergizes with PD 1 blockade in OXPHOS high dependency LC BrM PDOs, highlighting how a PDO platform can translate combined metabolic-immune vulnerabilities into testable therapeutic strategies (Duan et al., 2024).

Methodologically, PDOs offer a highly programmable system for seeing and controlling metabolic heterogeneity. Nguyen et al. integrated metabolic fluorescent reporters, single cell lineage tracking, and machine learning (Nguyen et al., 2025),to simultaneously follow cell state transitions and glycolytic flux within tumor organoids. They found that highly glycolytic cells export lactate to less glycolytic cells, which then recycle lactate to sustain stemness. Lactate metabolism enhances histone acetylation and activates MYC driven dedifferentiation programs, thereby causally linking metabolic state to stemness and differentiation trajectories within a single PDO. Duan et al. further built a large scale multiomics map spanning genomic, transcriptomic, proteomic, and metabolomic layers from paired primary lung cancers and brain metastases, and then used brain metastasis derived PDOs and mouse models to validate the efficacy of targeting mitochondrial OXPHOS, for example with gamitrinib, in combination with immunotherapy. This workflow enables metabolic vulnerabilities inferred from multiomics to be functionally traced back and perturbed in PDOs (Duan et al., 2024).

In summary, these studies underscore distinctive advantages of organoids for metabolic heterogeneity research. Their three-dimensional architecture and preservation of patient specific molecular features provide a scaffold for complex metabolic niches. At the same time, coupling PDOs with fluorescence lifetime imaging microscopy, flux analyses, multiomics integration, and genetic or pharmacological perturbations makes it possible to close the loop on a single platform, from drawing a metabolic landscape, to pinpointing pathology relevant subtypes, to validating targets *in situ*. This constitutes an especially powerful experimental arena for understanding and exploiting intratumoral metabolic heterogeneity at the molecular level.

#### 3.2.2 Immune evasion

From a behavioral standpoint, immune evasion is another major axis of intratumoral heterogeneity. Across tumors, immune contexture is often grouped into three archetypes, Immune-inflamed, Immune-excluded, and Immune-desert (Chen and Mellman, 2017; Arrieta et al., 2023). They differ systematically in how deeply effector T cells infiltrate, how intact antigen presentation remains, and how immunosuppressive circuits are assembled. Immune-inflamed regions contain abundant CD8^+^ T cells but often show high expression of exhaustion associated markers such as PD-1, PD-L1, and CTLA-4. Immune-excluded regions feature T cells that accumulate in the stroma or at the invasive front yet fail to penetrate the tumor parenchyma. Immune-desert regions show little to no adaptive immune infiltration. Recent multiomics and spatial omics studies further indicate that these landscapes are not restricted to differences between patients or lesions, but can coexist as patchwork patterns within a single tumor section, reflecting differential coupling among clonal lineages, vascular and metabolic niches, and cell intrinsic signaling programs (Tufail et al., 2025).

Operational definitions of immune exclusion vary across studies, but in general it refers to a situation in which effector immune cells are present in the tumor site yet cannot enter the tumor parenchyma and therefore cannot effectively engage cancer cells. PDOs provide a distinctive platform to reconstruct these immune evasion programs under controlled conditions. A range of 3D organoid and immune cell coculture formats has been developed, including direct coculture, Transwell systems, and microfluidic organoid-on-chip platforms. These approaches can incorporate autologous or allogeneic TILs, PBMCs, NK cells, macrophages, and dendritic cells while preserving patient specific genomic backgrounds, enabling systematic readouts of infiltration depth, exhaustion trajectories, and cytokine networks (Quintard et al., 2024; Major et al., 2024; Kastenschmidt et al., 2024; Zhou et al., 2023; Wang et al., 2024b; Chen X-Y. et al., 2025; Dong et al., 2022).

More importantly, organoid platforms allow clonal lineages, signaling pathways, and immune phenotypes to be linked with causal evidence. In esophageal squamous cell carcinoma, Ko et al. generated 32 mouse esophageal organoid genotypes carrying distinct combinations of driver alterations and used CRISPR Cas9 to sequentially inactivate TP53, CDKN2A, NOTCH1, and other key genes. Combined loss of TP53, CDKN2A, and NOTCH1, the PCN triple mutant, not only promoted squamous carcinoma like morphological conversion but also remodeled an immunosuppressive TME through upregulation of chemokines such as CCL2. In transplantation models and single cell profiling, tumors derived from PCN organoids were associated with enrichment of exhausted like CD8^+^ T cells and M2 like macrophages (Ko et al., 2023). This work effectively establishes a workflow from engineered organoids to *in situ* or xenograft growth to single cell or spatial transcriptomics, directly connecting driver gene combinations with chemokine programs and immune evasion patterns.

PDOs can also be integrated with high dimensional immunoprofiling and behavioral assays to identify druggable nodes within immune evasion networks. In high grade serous ovarian cancer, Wang et al. identified a JAG2^+^ subset of tumor associated neutrophils and showed, using multiplex immunohistochemistry, single cell RNA sequencing, and PDO coculture, that these cells express high PD-L1 and other suppressive molecules, drive CD4^+^ T cells toward effector Treg differentiation, and markedly blunt responses to anti PD-1 therapy (Wang C. et al., 2025). Notch inhibition with LY3039478 or JAG2 neutralizing antibodies restored T cell effector function and enhanced PD-1 blockade in both PDO and mouse models, suggesting that the JAG2^+^ TAN and eTreg circuit is an actionable node. Similarly, Kan et al. reviewed how gastric cancer PDOs can be used to dissect immune evasion in the TME, including regulation of PD-L1 expression, microbiome associated inflammation, and rational combinations with immune checkpoint blockade. They highlighted that PD-L1 upregulation driven by the HER2 PI3K AKT mTOR axis can be mechanistically resolved in gastric cancer PDO models and used to evaluate synergy between anti HER2 agents and immune checkpoint inhibitors (Kan et al., 2025). Within a single patient or a single pathological subtype, immune evasion is therefore not a single program but a set of immunosuppressive modules that different clones can deploy in different combinations, and PDO platforms enable these modules to be parsed and their vulnerabilities tested.

From the perspective of intratumoral heterogeneity, combining organoid and immune coculture with microfluidics, vascularization, or matrix engineering makes the Immune-inflamed, Immune-excluded, and Immune-desert states more than static labels on pathology sections. Instead, they become phenotypes that can be tracked over time and manipulated with causal control within the same PDO context. This capacity to dissect immune evasion programs under a shared genetic background and a tunable microenvironment makes PDOs an effective experimental bridge from molecular pathways to immune phenotypes to therapeutic vulnerabilities, and it lays the methodological groundwork for organoid guided combination immunotherapy informed by immune landscape stratification.

#### 3.2.3 Migration and invasion programs

Only a minority of tumor cells at the primary lesion’s leading edge acquire sufficient motility, extracellular matrix remodeling capacity, and stress tolerance to truly breach the basement membrane, migrate along vessels or perineural sheaths, and ultimately establish distant colonies. Classic EMT studies and recent reviews have reframed this process from a single linear epithelial to mesenchymal transition into a multistate continuum. Fully EMT, partial EMT (pEMT), and diverse hybrid epithelial and mesenchymal states can coexist within the same tumor and support distinct invasion modes, including single cell dissemination, collective migration, and budding like invasion (Allgayer et al., 2025; Dong et al., 2019). Spatial omics and longitudinal sampling further indicate that the tumor front is rarely a uniform EMT region. Instead, it often comprises highly invasive leading-edge cells, an intermediate transition zone, and relatively quiescent core cells arranged along a gradient. These compartments differ in integrin and FAK signaling, Rho GTPase circuitry, extracellular matrix fiber reorganization programs, and interaction patterns with immune and vascular elements, together forming a basic architectural unit of invasion heterogeneity (Ren et al., 2023; Zeng and Jin, 2022).

The distinctive value of tumor organoids for migration and invasion lies in their ability to preserve patient specific genomic backgrounds while reconstructing structured leading edge and core heterogeneity within a controlled 3D matrix. In PDAC, Huang et al. used collagen embedded PDOs to demonstrate two stable invasion modes that occur across patients and even within individual organoids, a mesenchymal like single cell pattern and a collective like pushing pattern. They further linked SMAD4 loss to collective invasion, and showed that restoring SMAD4 or perturbing downstream Rho GTPase signaling can reroute invasion trajectories at the organoid level. By retaining patient specific driver alterations while providing resolvable invasion morphologies and quantifiable genetic or pharmacologic perturbation effects on a single platform, organoids translate morphological heterogeneity at the invasive front into molecular programs that can be tested for causality (Huang et al., 2020).

Regarding dynamic spatial organization, Hwang et al. utilized microfluidic mammary tumor organoids with controlled oxygen tension, SDF1 gradients, and interstitial flow to investigate leader cell emergence. They demonstrated that K14^+^ leader cells are initially scattered stochastically throughout the organoid rather than being pre-positioned. Driven by hypoxia and chemotactic cues, these cells polarize along the CXCR4-SDF1 axis and DDR2-mediated mechanosignaling to accumulate at the leading edge, forming a “leader band” with enhanced traction and ECM remodeling capacity that drives collective migration (Hwang et al., 2019). Complemented by further dissection of the leader-follower interface (Hwang et al., 2023), these studies redefine the invasive front as a functional micro-niche that self-organizes under environmental gradients.

Organoids can also incorporate key stromal components to dissect multicellular interactions at the invasive front. In PDAC, Sun et al. identified endothelial like CAFs (eCAFs) as a stromal subset that promotes vascular mimicry and dissemination, and showed in PDO CAF coculture and organoid on chip systems that eCAFs build vessel like lumens around organoids and markedly enhance tumor budding and infiltration. Targeting eCAF specific pathways selectively suppressed this invasion route (Sun et al., 2025). In brain tumor models, Tamborini et al. used cortex organoid based assembloids to show that Connexin-43^+^ large extracellular vesicles derived from glioblastoma remodel astrocytic networks and enhance invasion, whereas Connexin 43 blockade significantly reduced invasion depth within organoids (Tamborini et al., 2025). Shard et al. further validated in patient derived glioblastoma explant organoids that leading edge enriched GABA_A_R signaling can be targeted, with antagonists selectively attenuating leading edge expansion (Shard et al., 2025). These models indicate that organoid platforms can capture and manipulate CAF tumor, neural tumor, and vesicle mediated communication circuits that are characteristic of the true invasive front.

In addition, organoids can bring tumor invasion and immune infiltration into the same experimental framework. Ou et al. developed melanoma PDO models in Matrigel and collagen to assess responses to PD 1 blockade and to quantify TIL chemotaxis and migration, respectively. They found that sensitivity to immune checkpoint therapy correlates with the depth of TIL infiltration in three dimensional collagen, enabling a direct quantitative view of the spatial mismatch between outward tumor invasion and inward immune entry (Ou et al., 2023). In parallel, Yan et al. proposed a distinctive invasive zone in liver cancer based on spatiotemporal omics, enriched for pEMT, immunosuppression, and vascular remodeling signals. Organoids can serve as an experimental fulcrum to decompose such programmatic invasion features into controllable modules (Yan et al., 2025).

These capabilities position PDOs and organoid models not only to recapitulate invasion heterogeneity, but also to convert it into testable and actionable therapeutic vulnerabilities.

#### 3.2.4 Proliferation dynamics

Within intratumoral heterogeneity, proliferative dynamics manifest as systematic differences among tumor subpopulations in cell cycle progression, tolerance to replication stress, and the capacity to enter dormancy and subsequently resume growth. Updated frameworks of the hallmarks of cancer treat sustained proliferative signaling and replicative immortality as core functional capabilities, and adaptation to replication stress is increasingly viewed as a key mechanism that drives diversification of these subpopulations (Hanahan, 2022). Across many tumor types, highly proliferative subsets often feature strengthened E2F and Myc programs, constrained nucleotide supply, and increased reliance on DNA damage repair, creating heightened sensitivity to checkpoint dependencies such as ATR-CHK1 or WEE1-CDK1/2 (Da Costa et al., 2023).

Using multiomics analyses in hepatocellular carcinoma, Jia et al. derived a replication stress signature and stratified patients into RS high and RS low groups. RS high tumors were enriched for DNA damage response and cell cycle pathways, had poorer prognosis, yet showed greater chemosensitivity. Importantly, RS high HCC displayed marked synergy with combined WEE1 inhibition and oxaliplatin, whereas RS low tumors were relatively tolerant, indicating that replication stress level itself constitutes an actionable dimension of behavioral heterogeneity (Jia et al., 2025). This framework is particularly relevant in the PDO setting because PDOs can preserve such replication stress linked dependencies within patient specific genomic backgrounds.

A second advantage of PDOs is that they enable high spatiotemporal quantification of proliferative kinetics in a true 3D context. Wu et al. developed a 96-well microgel platform for long term imaging to support long range imaging of PDOs in a thin 3D microenvironment. Following gemcitabine treatment, overall volumetric growth may transiently plateau, yet single organoid tracking revealed that a small fraction of high regrowth subpopulations rapidly restarted proliferation after drug withdrawal, producing a late rebound inflection point (Wu et al., 2022). This observation suggests that post treatment relapse can originate from rare subsets with superior replication recovery capacity, a feature that becomes apparent only through three dimensional dynamic readouts.

PDOs can also be used to validate how specific molecular events shape high proliferative phenotypes. In triple negative breast cancer, Song et al. reported that a circCAPG encoded peptide, CAPG 171aa, enhances proliferation and migration via the MEKK2-MEK1/2-ERK1/2 pathway. In patient derived TNBC PDOs, circCAPG knockdown significantly reduced organoid growth rates (Song et al., 2023). This aligns mechanistic signaling nodes with behavioral readouts in patient relevant contexts, positioning PDOs as a useful platform to dissect coupling between proliferative drive and invasive potential.

At the same time, PDO panels are increasingly used to screen for targets that reflect dependence on proliferative transcriptional programs. In ER^+^ breast cancer, Soosainathan et al. found that most endocrine and palbociclib resistant PDOs were highly sensitive to the CDK9 inhibitor AZD4573, whereas a minority remained resistant, indicating that even within a shared molecular subtype there exist behavioral subgroups with distinct transcriptional and proliferative dependencies (Soosainathan et al., 2024). In PDX models, combining AZD4573 with palbociclib or fulvestrant reversed growth in a subset of resistant tumors, directly supporting the translational vulnerability of a high proliferation and high transcriptional dependency state.

In aggregate, the behavioral-axis view emphasizes that intratumoral heterogeneity is primarily expressed through coupled and plastic functional states rather than static genetic partitions alone. By providing quantitative phenotypes and programmable perturbations within a patient-specific context, PDO platforms turn these state modules into experimentally testable coordinates, thereby enabling mechanism-linked interpretation and rational intervention against within-tumor adaptive diversity.

## 4 Intertumor heterogeneity revealed by tumor organoids

### 4.1 Interpatient heterogeneity

Interpatient heterogeneity in cancer arises from a complex mixture of factors. It reflects tumor intrinsic differences, including mutational landscapes, copy number alterations, and transcriptional programs, and is also shaped by the microenvironment and host biological context (Micoli et al., 2025; Diao et al., 2024; Ren et al., 2024; Pei et al., 2025). These differences often translate into individualized trajectories of treatment response and disease progression.

Breast cancer provides a clear example of interpatient heterogeneity. Based on molecular features, it is commonly classified into five subtypes, illustrating how molecular variation can be organized into a reusable subtype framework that feeds into clinical decision making (Song et al., 2023; Perou et al., 2000). Such differences can be functionally operationalized on PDO platforms as measurable variation in therapeutic response. The head and neck tumor organoid biobank HNOB, for instance, showed phenotypic heterogeneity in *ex vivo* radiotherapy response across TP53 and HPV16 associated subtypes, and this variability correlated with individual outcomes and relapse risk (Issing et al., 2025). Similarly, a sarcoma organoid biobank comprising 44 organoid lines spanning multiple subtypes recapitulated molecular and cellular diversity and enabled drug screening and prediction of immune responses, including rapid assessment of CAR T cell activation and cytotoxic capacity (Ma et al., 2026). In pediatric glial tumors, Deligne et al. built a biobank and, using multiomics profiling together with staining of key clinical marker proteins, targeted sequencing, and variant allele frequency analyses, showed that organoids can longitudinally preserve clinically relevant phenotypic features of the parental tumors, offering reusable material for cohort level investigations (Deligne et al., 2025).

Beyond coding mutations, interpatient variation can also reside in the usage of distal regulatory elements and 3D chromatin architecture. Organoids provide an effective window into these patient specific epigenoregulatory programs. In malignant rhabdoid tumors, Liu et al. analyzed organoids from multiple patients alongside matched tumor tissues to map how SMARCB1 loss remodels regulatory landscapes. They found that different patients could use distinct sets of distal super enhancers that loop to the MYC promoter, thereby driving MYC hyperactivation. This enhancer-promoter configuration was well preserved in PDOs and could be supported by single cell RNA and Assay for Transposase-Accessible Chromatin multiomics from the primary tumors (Liu et al., 2023). This work highlights that, even in tumors with relatively low mutation burdens, intertumor heterogeneity may be carried predominantly by epigenetic regulatory divergence, and PDOs can stably preserve such differences and enable mechanistic dissection.

In small patient cohorts tested in parallel, PDOs have already enabled molecular features to be mapped onto drug response phenotypes at the individual case level. In glioblastoma explant organoids from seven patients, Jacob et al. tested standard of care, temozolomide plus radiotherapy, alongside multiple targeted agents and observed pronounced variability in responses across patients. Sensitivity patterns aligned with molecular features such as NF1 status and EGFR pathway activity, and in some cases *in vitro* responses showed preliminary concordance with post operative imaging changes, suggesting the potential of PDO panels to map *ex vivo* drug sensitivity onto clinical response (Jacob et al., 2020). In HR^+^ and HER2^+^ breast cancer, Liu et al. molecularly profiled 211 patients and established matched PDOs, showing that distinct molecular subtypes exhibit differential sensitivity to pathway specific inhibitors. These data indicate that multi patient PDO panels can functionally read out subtype linked pathway dependencies and connect transcriptional programs to therapeutic vulnerabilities (Liu C et al., 2025).

Larger scale studies further underscore the systematic value of PDOs for biomarker discovery and patient stratification. Yang et al. established a primary liver cancer PDO biobank spanning three pathological subtypes, hepatocellular carcinoma, intrahepatic cholangiocarcinoma, and combined hepatocellular cholangiocarcinoma, and successfully recapitulated the distributions of key mutations, CNAs, and transcriptomic subtypes reported in public cohorts. Using 255 PDO lines with matched RNA seq and drug response profiles, they applied machine learning to build expression based predictive models for multiple first line targeted therapies, and the predictions showed reasonable concordance with observed clinical benefit in an independent organoid cohort and in a small patient set. These results highlight the potential of PDO platforms for extracting biomarkers that can inform stratification decisions (Yang et al., 2024). In pancreatic ductal adenocarcinoma, Li et al. integrated exome and transcriptome sequencing, screening of 111 commonly used anticancer agents, and matched patient outcomes to show that PDOs together with companion *in vivo* models preserve interpatient molecular variation while capturing corresponding heterogeneity in drug sensitivity (Li et al., 2025). They further identified UGT1A10 from cross patient drug response patterns as a key determinant of differential responses to multiple chemotherapies, completing a closed loop path that moves from a multi patient PDO panel to a metabolic gene driving functional heterogeneity and then back to validation in clinical cohorts. Notably, by incorporating immune cell coculture, the study extended interpatient comparisons to immunotherapy and cell-based therapy response assessment, providing an actionable preclinical platform for individualized response prediction in PDAC, a prototypical immune cold tumor.

Beyond passively reading out differences, PDOs can also support mechanistic stratification and therapy matching within an explicit molecular subgroup framework. Hasselluhn et al. defined a PDAC subgroup characterized by SMAD4 loss and high NFATc1 expression in public cohorts, and then showed in mouse PDAC models and PDOs that SMAD4 deficiency promotes assembly of an NFATc1/SMAD3/cJUN complex that upregulates RRM1 and RRM2 to maintain nucleotide pools. This creates competition with activated gemcitabine metabolites and drives resistance. In this subgroup, MEK inhibition disrupted the complex and resensitized organoids to gemcitabine, illustrating the selective value of PDOs for validating subgroup specific combination strategies (Hasselluhn et al., 2024). Sun et al. established an oral mucosal melanoma PDO biobank and, combining drug response profiling with immune coculture, demonstrated substantial interpatient variability in responses to RTK inhibitors, CDK4/6 inhibitors, and anti PD 1 regimens. They further proposed a molecular subtype guided framework for combination therapy and stratification (Sun et al., 2023), highlighting the advantage of PDOs in rare tumors and immunotherapy contexts for linking molecular subtypes to functional vulnerabilities and rational combination design. In the nanomedicine arena, Boix Montesinos et al. further showed that PDOs can translate interpatient variation in metabolic and enzymatic parameters into functional biomarkers and support individualized selection of specific nanotherapeutics (Boix-Montesinos et al., 2025). Similar approaches have been repeatedly applied in lung cancer (Duan et al., 2024; Oh et al., 2024), cervical cancer (Lin et al., 2023), colorectal cancer (Mazzeschi et al., 2022), ovarian cancer (Micoli et al., 2025), and other solid tumors to validate subtype associated differences in drug sensitivity, reinforcing the generalizability of a molecular stratification plus PDO functional validation strategy.

Conventional epithelium-only PDO systems preserve many tumor-cell-intrinsic determinants of intertumor heterogeneity and can report cell-intrinsic responses to selected cues, yet they lack intact immune and stromal ecosystems and therefore have limited fidelity for heterogeneity that is realized through immune composition, stromal remodeling, and niche-level spatial constraints. Accordingly, organoid formats that retain immune components or engineer microenvironmental context provide a critical route to recover these missing heterogeneity dimensions. Dong et al. cultured PDOs in suspended alginate gelatin hydrogel capsules to better approximate the liver microenvironment. They detected the presence of hepatocyte growth factor (HGF), together with components such as cancer associated fibroblasts (CAFs), and vascular endothelial cells (VECs), and the platform recapitulated interpatient heterogeneity in drug screening assays (Dong et al., 2022). Song et al. provided evidence that tissue derived extracellular matrix hydrogels can improve preservation of heterogeneity in organoid culture. They prepared a uterine cervix extracellular matrix (UCEM), hydrogel from peritumoral cervical tissue and used it to replace Matrigel for culturing cervical squamous cell carcinoma organoids (Song et al., 2024). RNA seq suggested that UCEM based culture increases transcriptomic similarity to the parental tumors, enriches CSCC relevant signaling programs, and yields clinically relevant drug resistant phenotypes. Du et al. generated 33 personalized three dimensional bioprinted gastric cancer models using patient derived gastric cancer tissues and retained a degree of TME components. The models displayed pronounced patient to patient differences in architecture, ECM dependent behaviors, and the proportions of immune and stromal cells, underscoring the contribution of microenvironmental context to interpatient variation (Du L. et al., 2025). In high grade serous ovarian cancer, Wang et al. used a short term organoid system that retains CD45^+^ immune cells to functionally read out how interpatient differences in immune pathways drive heterogeneous responses to PD 1 blockade, and to provide testable rationales for combination interventions (Wang C. et al., 2025).

**FIGURE 2:**
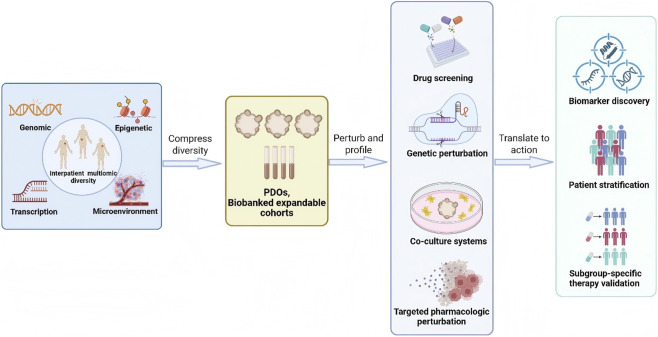
Patient derived organoid biobanks translate interpatient molecular diversity into functional phenotypes and actionable stratification. The image was created by BioRender.

As summarized in Figure 2, the value of PDO biobanks for intertumor heterogeneity research can be distilled into two points. First, at the multiomics level, they compress interpatient differences in the genome, transcriptome, and even epigenoregulatory programs into reproducible and expandable experimental cohorts. Second, through drug screening, genetic and pharmacologic perturbations, and coculture based readouts, they translate molecular stratification into quantifiable functional phenotypes, thereby supporting biomarker discovery, patient stratification, and validation of subgroup specific therapeutic strategies (Issing et al., 2025; Yang et al., 2024; Ringnalda et al., 2025; Farin et al., 2023).

### 4.2 Intrapatient, interlesional heterogeneity

Within a single patient, tumors arising at different anatomical sites, and even multiple lesions within the same organ, are rarely a homogeneous whole. Rather, they form a multicentric system in which a shared ancestral clone carrying early driver events undergoes branched evolution under distinct spatial niches, tissue microenvironments, and therapeutic pressures (Kim et al., 2022). Clinically, this divergence often manifests as mixed responses to systemic therapy, with some lesions shrinking while others remain stable or progress. Such lesion level molecular and functional heterogeneity has become a central challenge for precision oncology.

Cross organ metastasis is a major source of interlesion heterogeneity. In pancreatic cancer, Pei et al. integrated spatial transcriptomics, inferred CNV, and PDO coculture assays to show that lineage composition and stromal interactions differ markedly across metastatic organs (Pei et al., 2025). In their study, liver and peritoneal metastases were often enriched for basal like cells, whereas lung metastases were predominantly classical like. Even synchronous metastases within the same organ could display distinct lineage mixtures. Notably, when these PDOs were cocultured with fibroblasts, basal like PDOs more readily recruited α SMA^+^ myCAFs, whereas classical like PDOs showed little such capacity. Functionally reconstructing these microenvironmental interaction differences in organoid systems helps explain why metastases vary in proliferation, invasion, and drug sensitivity, and provides a foundation for studying lesion level functional heterogeneity.

In more extreme spatial niches, lesion specific evolutionary trajectories can be even more pronounced. In breast cancer leptomeningeal metastasis, Fitzpatrick et al. profiled cerebrospinal fluid cfDNA and established CSF derived PDOs to map early branched relationships among primary tumors, extracranial metastases, and leptomeningeal lesions. Leptomeningeal metastases were shown to diverge early from trunk clones and to be enriched for alterations in genes involved in adhesion junctions and cytoskeletal regulation, including CDH1, CTNNA1, and ARHGEF10, yielding an E cadherin loss phenotype reminiscent of lobular carcinoma that was typically absent in extracranial lesions. Importantly, CSF PDOs functionally recapitulated resistance of leptomeningeal clones to intrathecal methotrexate as well as their tropism toward leptomeningeal and endocrine related environments, indicating that organoids can capture not only interlesion molecular divergence but also CNS specific therapeutic liabilities in functional form (Fitzpatrick et al., 2023).

Beyond organ specific microenvironmental differences, broader evolutionary stratification within a patient can also generate interlesion divergence. Lahtinen et al. analyzed 510 multi-region samples from 148 high grade serous ovarian cancer patients and identified three evolutionary states dominated by distinct signaling pathways. These states differed significantly in clonal architecture and chemotherapy response, and primary lesions were enriched for private clones, consistent with greater evolutionary distinctiveness. PDOs derived from tumors with high PI3K and AKT activity showed enhanced sensitivity to multiple PI3K inhibitors, providing functional support for a causal link between evolutionary state, pathway activity, and targetable vulnerability. This illustrates how PDOs can translate lesion level pathway differences into precise drug response patterns (Lahtinen et al., 2023).

Notably, interlesion heterogeneity is not limited to primary versus metastatic relationships. Multiple lesions within the same organ can undergo partially independent evolution. In multifocal primary prostate cancer, Kang et al. established matched PDOs for each lesion using mirror image biopsies and found pronounced divergence across lesions in genomic and transcriptomic profiles as well as drug sensitivities (Kang et al., 2025). PDOs from some lesions were highly sensitive to RTK inhibitors, whereas others were fully resistant. The authors further built predictive models from lesion tissue RNA seq that could forecast therapeutic response even without PDOs. These findings indicate that multifocal tumors comprise coexisting, independently evolving units, and treatment sensitivity cannot be reliably inferred from a single biopsy.

Extending the spatial view to the circulation reveals another form of interlesion heterogeneity shaped by disseminated clones. Circulating tumor cell derived organoids (CTCDOs) retain the genomic and phenotypic features of their source clones and offer the advantage of repeatable sampling through liquid biopsy, enabling noninvasive tracking of clonal diversification and resistance evolution during dissemination (De Angelis et al., 2022; Pan et al., 2025). For example, De Angelis et al. reported in a colorectal cancer model that CTCDOs, compared with PDOs and xenograft derived organoids, displayed higher stemness, stress tolerance, and invasive potential, along with distinct pathway dependencies and drug sensitivities. Whereas xenograft derived organoids were primarily dependent on EGFR signaling, CTCDOs were markedly sensitive to the Survivin inhibitor YM155. By systematically comparing the functional and pathway dependencies of related clones in organoid systems, this work demonstrates how heterogeneity of disseminated clones can be amplified in drug response phenotypes (De Angelis et al., 2022).

A key implication is that a single biopsy or a single PDO line may not adequately represent the actionable heterogeneity within an individual patient. Lesion-resolved organoid panels, complemented by liquid biopsy-derived models such as ctDNA-informed monitoring and circulating tumor cell-derived organoids, provide a practical route to distinguish vulnerabilities that are shared across lesions from those that are lesion restricted. This framework supports prioritization of strategies that target trunk liabilities or rationally combine agents to cover divergent branches. More broadly, this lesion-level functional mapping motivates a testable hypothesis for precision oncology, namely that response and resistance are emergent properties of a multi-lesion system rather than attributes of a single dominant clone.

## 5 Temporal heterogeneity revealed by tumor organoids

Temporal heterogeneity refers to the continuous remodeling of molecular and phenotypic features of a patient’s tumor over the course of disease. It encompasses dynamic changes across multiple layers (Ko et al., 2023). This process is jointly driven by intrinsic evolutionary forces, such as stochastic clonal drift and clonal competition, and by extrinsic pressures, including strong treatment imposed selection, differences in tissue niches, and systemic host responses (Mathur et al., 2024). In clinical practice, direct observation of temporal heterogeneity remains highly constrained. Most patients undergo a single tissue biopsy at diagnosis. Even when resampling is performed at progression or relapse, it typically yields only one or two additional time points, often from different anatomical sites, thereby entangling spatial and temporal effects. In recent years, longitudinal liquid biopsy coupled with mathematical modeling of ctDNA has begun to reconstruct trajectories of clonal fractions over time and has value for predicting resistance and relapse, but it still lacks cell level functional readouts and controllable perturbation capacity (Dusetti et al., 2025).

Organ specific studies further emphasize the importance of the temporal dimension. For example, work on spatiotemporal heterogeneity in hepatocellular carcinoma has shown that, during progression and treatment, the invasive margin and tumor core undergo dynamic reorganization in driver mutations, immune infiltration lineages, and angiogenesis related signaling. These time dependent changes are strongly associated with formation of invasive regions and recurrence (Yan et al., 2025). Similarly, studies in breast cancer and pancreatic ductal adenocarcinoma increasingly treat temporal heterogeneity as a key molecular basis of recurrence, resistance, and metastasis rather than a static terminal state (Migliore et al., 2025; Dusetti et al., 2025; Gu et al., 2024). Longitudinal investigations using PDOs indicate that organoid models can preserve and reveal molecular diversity that continues to evolve over time. Single cell RNA sequencing and multiomics analyses show that organoids maintain diverse sub-clonal populations and that gene expression and mutational patterns can shift during long term culture or under drug exposure (Zhao et al., 2021). For instance, the bladder cancer PDO biobank established by Minoli et al. converted patient samples spanning stages and grades into a 3D system that can be expanded long term and manipulated pharmacologically (Minoli et al., 2023). More importantly, while largely preserving genetic and phenotypic features of the parental tumor, PDOs continue to undergo clonal competition, adaptation, and selection in culture. As a result, they are naturally suited to serve as experimental evolution platforms for reconstructing temporal heterogeneity (Thorel et al., 2024; Esposito et al., 2024; Tao et al., 2025).

**FIGURE 3:**
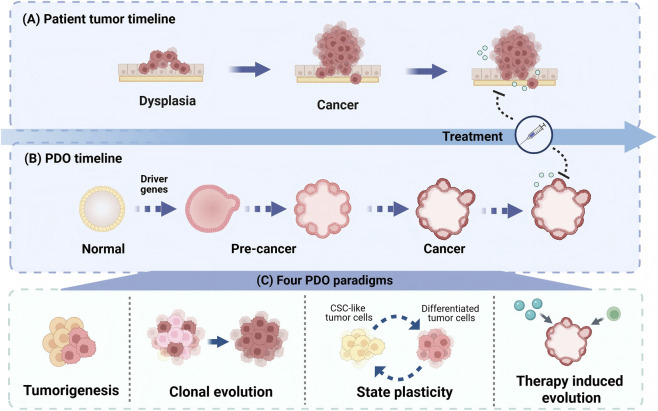
Organoid based framework for interrogating temporal heterogeneity. **(A)** Simplified patient tumor timeline from dysplasia to cancer and subsequent treatment exposure. **(B)** Corresponding PDO timeline aligned to key clinical stages and amenable to controlled perturbation and longitudinal sampling. **(C)** Four complementary PDO paradigms that operationalize time and link phenotypic readouts with longitudinal molecular profiling. The image was created by BioRender.

Methodologically, organoid based systems enable temporal heterogeneity to be interrogated beyond sparse clinical anchor points by pairing patient level disease course with controllable *ex vivo* timelines and longitudinal molecular profiling. As summarized in Figure 3, we organize organoid enabled temporal analyses into four complementary paradigms.

### 5.1 Natural history and tumor initiation: a timeline from normal organoids to precursor lesions

At the level of natural history, temporal heterogeneity is reflected not only in shifts of clonal lineages but also in progressive reprogramming of tumor epithelium along distinct lineage trajectories and in co evolution with the immune microenvironment. Models built from genetically engineered normal organoids make these early events tractable and precisely reconstructable *in vitro*. Ko et al. used CRISPR Cas9 to systematically delete different combinations of genes that are frequently mutated in esophageal squamous cell carcinoma (ESCC) in mouse esophageal organoids, generating 32 engineered organoid lines and profiling lineage and pathway changes by single cell transcriptomics (Ko et al., 2023). They showed that triple loss of Trp53, Cdkn2a, and Notch1 is sufficient to drive ESCC like lineage diversity and heightened plasticity, establishing this combination as a key initiating event for ESCC.

Karlsson et al. started from TP53 deficient mouse gastric epithelium and established a time series of organoids derived from distinct stages, spanning normal glands, precursor lesions, early cancer, and advanced lesions. By long term passaging with sequencing at multiple time points, they reconstructed the premalignant evolutionary route driven by TP53 inactivation. The data indicated that evolution in the TP53 deficient background is not fully stochastic but proceeds along a limited set of constrained clonal trajectories, accompanied by stage dependent activation and selection of pathways such as Wnt, TGF β, and DNA damage response programs (Karlsson et al., 2023). This type of work demonstrates that building matched organoids across evolutionary stages and extending the *in vitro* timeline can resolve the ordering of driver events and expose stage specific vulnerabilities.

Organoid studies of glioblastoma initiation (Sojka et al., 2025) and of tumorigenic conversion from developmental aberrations (Min et al., 2022) support a similar picture. By generating matched organoids across successive stages and prolonging the *in vitro* timeline, these models help define the sequence of key driver events and stage specific liabilities, capturing temporal heterogeneity along the depth axis from normal tissue to precursor lesions to invasive cancer.

At later stages of progression, temporal heterogeneity can also manifest as continued selection of specific early malignant subpopulations that ultimately shape relapse risk. In high grade meningioma, Huang et al. combined single cell transcriptomics with patient derived meningioma organoids and identified a SULT1E1^+^ tumor cell subpopulation enriched specifically in WHO grade II and III and in recurrent samples. Pseudotime analysis positioned this population on an early branch of the lineage trajectory, consistent with sustained positive selection of an initiating subpopulation during malignant progression (Huang M. et al., 2023). This population was preserved in meningioma organoids established from intact tumor tissue. Organoids containing SULT1E1^+^ cells reproduced characteristic brain invasive behavior after orthotopic implantation, whereas the small molecule SRT1720 suppressed the SULT1E1^+^ population and increased radiosensitivity. Together, these studies indicate that combining engineered normal organoids with organoids derived from progressive lesions enables *in vitro* reconstruction of a natural history timeline spanning tumor initiation, precursor lesions, high grade progression, and relapse, while allowing identification of early actionable molecular events and high-risk subpopulations at each stage.

### 5.2 Longitudinal PDO series: clonal evolution along real world treatment timelines

Compared with engineered models, pairing multi time point patient specimens with matched PDOs focuses directly on real world timelines of disease course and therapy.

In pediatric high grade glioma and ependymoma, Deligne et al. established a standardized workflow for generating pediatric tumor derived organoids and showed that these models retain parental tumor multiomics features and cellular state hierarchies, including OPC like, NPC like, AC like, and MES like programs, even after long term expansion and cryopreservation (Deligne et al., 2025). By drug testing paired PDOs derived from the same patient at diagnosis and at relapse, they found that sensitivity to agents such as larotrectinib and ONC201 declined markedly with treatment progression. They also captured canonical acquired resistance events, including NTRK2 G639R, in relapse derived PDOs, faithfully reflecting therapy driven temporal heterogeneity and acquired resistance.

Similarly, in rapidly progressive oral mucosal melanoma, Sun et al. generated an organoid biobank comprising 30 PDOs from 21 patients. Six paired sets were derived from sequential lesions arising within the same patient over a 2–6 months window, forming a within patient, stage matched longitudinal PDO series. Whole exome sequencing and phylogenetic reconstruction distinguished shared trunk driver events from evolution associated mutations and copy number changes that emerged only in later lesions. PDOs established at different time points also displayed pronounced differences in drug sensitivity and pathway dependencies (Sun et al., 2023).

These clinically anchored paired and multi time point PDO series provide discrete *ex vivo* snapshots for controlled comparisons of clonal architecture, cellular states, and drug liabilities across therapy linked time points.

### 5.3 Short timescale state plasticity: nongenetic temporal heterogeneity

Temporal heterogeneity is not only the product of slow clonal replacement. On timescales of days or even hours, reversible switching among stemness, EMT associated programs, and drug tolerant states constitutes an important form of nongenetic temporal heterogeneity. Such plasticity is often coordinated by signaling pathways including Wnt and NOTCH, TGF β, and by microenvironmental inputs.

Choi et al. built PDAC PDO based assembloids containing endothelial and immune components and found that differentiated ductal like tumor cells could be reprogrammed back to a CD24^+^CD44^+^EpCAM^+^ tumor initiating state within 7 days. This reversible transition depended on tumor cell JAG1, which activated an endothelial NOTCH WNT5B TGFB1 axis. Blocking JAG1 or NOTCH markedly suppressed this differentiated to initiating backflow, and high JAG1 expression was associated with poor prognosis (Choi et al., 2022).

For metabolic epigenetic driven temporal plasticity, Nguyen et al. used human colorectal cancer PDOs carrying fluorescent reporters of stemness and differentiation states as well as metabolic activity. Coupling continuous single cell live imaging lineage tracing with the CellPhenTracker machine learning pipeline, they mapped the time resolved co evolution of cell fate and metabolism within organoids (Nguyen et al., 2025). They found that lactate produced by differentiated cancer cells was preferentially taken up by cancer stem cells. Lactate increased mitochondrial respiration and acetyl CoA availability, promoted histone acetylation at the MYC locus, and activated MYC in a BRD4 dependent manner. This suppressed differentiation of CSCs and repeatedly drove dedifferentiation of differentiated cells back toward CSC states, reshaping clonal composition and proliferative dynamics.

Organoids can capture short timescale state cycling driven by microenvironmental cues and metabolic epigenetic circuits, resolving a form of nongenetic temporal heterogeneity that is difficult to disentangle in clinical specimens. This capability offers direct mechanistic insight into early drug tolerance and latent relapse risk.

### 5.4 Therapy induced temporal evolution: co evolution of resistance mechanisms

Organoids can be maintained long term under controlled conditions and exposed to sustained drug pressure with dense longitudinal sampling. This experimental evolution setup shifts temporal heterogeneity from a few clinical anchor points to a continuous trajectory, enabling reconstruction of the order in which resistance routes emerge, how multiple routes coexist, and when specific combinations become effective. Single cell and multiomics readouts then connect functional response shifts to state transitions and selection dynamics (Dagogo-Jack and Shaw, 2018).

Dilly et al. integrated longitudinal ctDNA from clinical trials of KRAS inhibition, a PDAC PDO cohort treated with the KRASG12D inhibitor MRTX1133, and KPC mouse models to map how resistance mechanisms appear continuously over time and become selected under targeted therapy (Dilly et al., 2024). Comparing pretreatment and progression ctDNA revealed stepwise emergence of multiple amplification events and acquired mutations across the treatment course. The PDO cohort provided a continuous response spectrum, ranging from high sensitivity to near primary resistance, and linked specific baseline molecular features to intrinsic resistance. *In vivo* models further showed that distinct amplification events, together with EMT programs and PI3K AKT mTOR signaling, arise sequentially as subclonal populations and expand. This longitudinal integration underscores a division of labor across modalities: liquid biopsy provides temporal clues, PDOs provide controllable functional readouts, and *in vivo* models capture ecological pressures and selection, together approximating co evolution of multiple resistance routes in real treatment settings.

When longitudinal biopsies are limited, stratified sampling across patients, disease stages, and treatment nodes can be used to reconstruct a latent temporal sequence, which can then be functionally validated and perturbed in PDOs. Huang et al. performed single cell transcriptomics on peritoneal lavage fluid and malignant ascites from 35 gastric cancer patients. They stratified samples into five groups, spanning benign controls, early gastric cancer, advanced gastric cancer, and pre and post peritoneal metastasis, including post systemic therapy, thereby constructing a stage aligned temporal cohort that tracks gastric cancer peritoneal metastasis and treatment progression (Huang X-Z. et al., 2023). Single cell analysis indicated that, with progression, monocyte derived dendritic cells progressively lost antigen presentation capacity and acquired proangiogenic features, while Tregs and exhausted like circulating T cells accumulated. After chemotherapy or PD 1 and PD L1 blockade, these immune subsets underwent further reprogramming in checkpoint expression and metabolic pathways. Using matched ascites derived PDOs and PDXs, the study further identified and validated a highly plastic tumor cell population that depends on autophagy and an mTORC1 linked paligenosis program and can switch between quiescence and high proliferation. Autophagy inhibition weakened this plastic clone and reduced peritoneal metastasis. This framework illustrates how single cell stage aligned temporal maps combined with PDO functional validation can translate therapy induced temporal heterogeneity into concrete time sensitive targets and rational drug combinations.

Beyond clonal and transcriptional programs, drug epigenome interactions are also continuously reshaped during treatment, helping explain state transitions that cannot be captured by mutations or transcriptional shifts alone. Dong et al. developed single cell EpiChem, scEpiChem, in colorectal cancer PDOs, enabling simultaneous measurement at single cell resolution of chromatin binding by small molecules, including the BET inhibitor JQ1, the CDK7 inhibitor THZ1, and doxorubicin, together with epigenomic signals such as H3K27ac and Assay for Transposase-Accessible Chromatin accessibility (Dong et al., 2024). By reconstructing an EMT trajectory across multiple exposure time points, Day 0, 3, and 5, they delineated the dynamic process of drug epigenome coupling over time. The results showed marked time dependent divergence in drug target occupancy and enhancer activity across subpopulations, providing a methodological paradigm for resolving chained temporal heterogeneity on a day scale, from drug pressure to epigenomic remodeling to state transition.

In addition, pseudo-time analysis can infer latent evolutionary sequences from static single cell snapshots of PDOs, helping flag potential pre resistant subpopulations in advance. Zhao et al. performed scRNA sequencing with pseudo-time analysis in hepatobiliary tumor PDOs, identifying clones with proliferative or metabolic advantages. In specific samples, they pinpointed a CD44^high^ stem like subpopulation that activated multiple resistance associated pathways and exhibited broad resistance to several TKIs. Ligand receptor analysis further suggested reinforcing signaling feedback between the metabolic advantage population and the resistance associated population (Zhao et al., 2021). When combined with longitudinal cohorts or paired pre and post treatment sampling, pseudotime based approaches may enable prospective identification of potential pre resistant clones with combined metabolic advantage and CSC like features before therapy initiation, informing time stratified combination regimens and dynamic monitoring strategies.

In brief, organoids convert dynamic processes that are otherwise observable only at a handful of clinical time points into an experimentally tractable timeline that can be continuously tracked in culture, dissected by multiomics profiling, and functionally tested through pharmacologic perturbation. Spanning natural history and tumor initiation, short timescale cycles of state plasticity, therapy induced evolution of resistance, and latent trajectories inferred by pseudotime, organoid platforms transform temporal heterogeneity from an abstract concept into measurable and controllable molecular programs. Clinically, this shift not only helps clarify the key events and their ordering that drive progression and relapse, but also provides unprecedented experimental support for developing time sensitive biomarkers and for designing adaptive therapies or stage stratified combination regimens.

## 6 Balanced appraisal of patient derived organoids in tumor heterogeneity research

PDOs provide an experimentally controllable platform for investigating tumor heterogeneity across intrapatient, interpatient, and temporal dimensions. From this perspective, the central question is which dimensions of heterogeneity they can reliably capture, under what conditions, and how their interpretive power can be strengthened through methodological integration.

### 6.1 Boundary conditions and methodological constraints of PDOs in modeling tumor heterogeneity

A primary limitation of PDOs lies in their incomplete reconstruction of the tumor microenvironment and higher order tissue architecture. Most PDOs grow as nonvascularized three dimensional epithelial structures and therefore lack a perfused vascular system, physiological mechanical forces, long range stromal organization, and stable immune compartmentalization (Guelfi et al., 2025; Sun et al., 2024; Pennel et al., 2024). As a consequence, gradients of oxygen, nutrients, metabolites, and drug exposure are determined mainly by diffusion and organoid size rather than by the coordinated vascular and tissue level dynamics that shape tumors *in vivo*. This means that PDOs are generally better suited to modeling cell intrinsic programs and short-range niche effects than to faithfully reconstructing the complex spatial stratification that spans tumor edges, cores, invasive fronts, and immune excluded regions.

An additional and often underappreciated constraint lies in sampling itself. Tumor heterogeneity is spatially uneven, whereas many PDOs are derived from a single biopsy or a limited tumor fragment (Minoli et al., 2023; Monteiro et al., 2022; Cioce et al., 2023). Under such circumstances, the resulting culture may selectively preserve only a subset of the original tumor ecosystem. This issue is especially important in tumors characterized by pronounced regional divergence, metastatic branching, or patchy therapeutic pressure. Thus, the interpretive value of a PDO depends not only on how faithfully it grows, but also on how representative the source material is.

Beyond limitations in spatial reconstruction and source representativeness, PDOs also face important challenges in capturing temporal heterogeneity. In principle, PDOs offer an attractive system for tracking clonal selection, lineage plasticity, and treatment induced state transitions. In practice, however, these possibilities remain constrained by limited sampling, culture associated selection pressure, and passage dependent remodeling. Because of clonal drift, altered growth competition, or adaptation to *ex vivo* conditions, late passage organoids may gradually diverge from the biological state of the parental tumor. In addition, although some progress has been made, longitudinally matched collections spanning baseline, treatment, and relapse remain relatively scarce in many cancer types (Kim et al., 2025). Therefore, PDO based analyses of temporal heterogeneity are most compelling when anchored to precisely defined clinical sampling time points.

### 6.2 Interpreting divergent findings across PDO studies

Differences across PDO studies do not necessarily indicate that the model itself is unreliable. In many cases, such differences reflect variation in what is being modeled, how it is cultured, and how it is analyzed. Specifically, divergent conclusions may arise from differences in tissue procurement, digestion procedures, extracellular matrix composition, culture media formulations, passage number, assay timing, and endpoint selection (Punzi et al., 2025). Therefore, even when two studies appear to address the same biological question, they may in fact be capturing different layers of heterogeneity, such as pre existing clonal diversity, transient cell state plasticity, or microenvironment dependent drug adaptation (Major et al., 2024; Cioce et al., 2023; Rodarte et al., 2024; Thorel et al., 2023; Betge et al., 2022).

For example, some studies emphasize the high genomic and transcriptomic concordance between PDOs and their parental tumors (Peng et al., 2025; Mo et al., 2022; Zou et al., 2022), whereas others report selective loss or enrichment of particular subclones during establishment and expansion (Farin et al., 2023; Chen et al., 2024). These observations are not necessarily contradictory, but may instead reflect differences in study design and analytical resolution. Bulk level comparisons may support an overall picture of fidelity, whereas single cell or single organoid analyses may reveal selective shifts within minority populations. Accordingly, interpretation of organoid based data should specify which biological layers are robustly preserved and which remain unstable.

The same logic also applies to studies of drug response. Apparent pharmacological discrepancies may arise because different platforms quantify different response readouts (Tebon et al., 2023; Lee et al., 2024; Xiang et al., 2024). In this sense, inconsistency across studies often reflects the fact that heterogeneity is being captured at distinct biological and technical levels.

### 6.3 Standardization in PDO research

One of the most immediate barriers to the broader use of PDOs in heterogeneity research is the lack of unified and standardized workflows (Kim et al., 2025). The success rate of PDO establishment and the outcomes of downstream experiments can both be strongly influenced by variation in sample acquisition and handling, tissue digestion procedures, culture media composition, extracellular matrix or scaffold materials, passaging schedules, cryopreservation and recovery conditions, and analytical pipelines. Without a higher degree of procedural consistency, it remains difficult to determine whether differences across studies reflect genuine biological divergence or laboratory specific technical effects.

Reproducibility is also challenged by the way responses are measured. Traditional detection methods often compress heterogeneous behaviors into a single endpoint and may therefore overlook the important distinction between cytostatic and cytotoxic effects or obscure subclone specific responses (Deben et al., 2024). The recent development of imaging based approaches and multilevel assays reflects the fact that organoid systems require response metrics capable of capturing growth dynamics, morphological diversity, and within culture variability (Tebon et al., 2023; Ramos et al., 2023). This issue is particularly important in heterogeneity research, where the central question is often not the average response of the culture, but the unequal responses of distinct subpopulations within it.

### 6.4 Enhancing the interpretive power of PDOs through multiomic and multimodel integration

Precisely because PDOs have clear boundary conditions, their value is maximized when they are embedded within integrated analytical frameworks rather than treated as stand-alone surrogates of tumors.

One important direction is the combined use of genomics, transcriptomics, epigenomics, and functional drug screening (Diao et al., 2024; Lee et al., 2025; Chen X-Y. et al., 2025; Jia et al., 2025; Wang G-Z. et al., 2025; Merrill et al., 2024; Li et al., 2024; Cho et al., 2023). For example, in liver cancer, Yang et al. established a biobank of 399 organoids from 144 patients and integrated histopathology, genomic characterization, and pharmacogenomic profiling, showing that inter- and intra-tumoral heterogeneity could be linked to clinically relevant drug response patterns within the same experimental system (Yang et al., 2024). In glioblastoma, Jacob et al. generated a patient-derived organoid biobank that preserved both inter- and intra-tumoral heterogeneity and further correlated mutational profiles with responses to selected drugs and CAR-T-cell therapy, thereby illustrating how molecular diversity can be functionally translated into therapeutic stratification (Jacob et al., 2020). A similarly integrative logic was applied in head and neck cancer by Issing et al., who established a molecularly and functionally characterized organoid biobank representing clinically relevant TP53-mutant and HPV16-driven subtypes, correlated radiotherapy responses with clinical data, and used genetically engineered organoids to test the direct contribution of major driver contexts to treatment phenotype (Issing et al., 2025). Collectively, these studies show that integrated PDO biobanks are especially valuable for heterogeneity research because they do not simply indicate whether an organoid is sensitive or resistant, but help define which driver contexts, cellular states, and regulatory programs correspond to specific therapeutic vulnerabilities.

Another major advantage of multiomic integration lies in improving the interpretability of PDO phenotypes. Morphological variation, growth behavior, and treatment response in organoids do not always directly reveal their underlying mechanisms, but when these phenotypes are analyzed together with matched molecular layers, they can be transformed into biologically meaningful state frameworks. For example, long-term ovarian cancer PDOs combined histology, copy-number analysis, transcriptomics, homologous recombination testing, and drug screening, showing that heterogeneous chemotherapy and PARP inhibitor responses could be interpreted in relation to genome instability and homologous recombination status (Thorel et al., 2025). In cholangiocarcinoma, integrated transcriptomic and pharmacologic analyses showed that the enhanced efficacy of DNMT and PARP inhibition was associated with inflammatory signaling, cell-cycle suppression, and senescence induction, thereby turning a drug-response phenotype into a mechanistically interpretable vulnerability state (Wang P. et al., 2025). In glioblastoma, sequencing-guided target selection together with organoid assays, single-cell RNA sequencing, and mass cytometry helped relate CAR-T efficacy to antigenic heterogeneity and immune phenotypes (Zhai et al., 2025). Moreover, when PDOs are combined with CRISPR-based perturbation, correlational findings can be extended into causal validation. In osteosarcoma, organoid profiling together with CRISPR screening identified ERCC6 as a determinant of cisplatin resistance and linked this phenotype to downstream splicing and PI3K/AKT-associated mechanisms (Xu et al., 2025). Studies of endothelial-like cancer-associated fibroblasts in pancreatic cancer further suggest that some aggressive phenotypes cannot be fully interpreted without incorporating stromal signaling, indicating where multiomic integration of PDOs still needs complementary niche-level models (Sun et al., 2025). The value of multiomic integration lies in rendering organoid phenotypes mechanistically interpretable by linking observable responses to their underlying biological states within the same experimental system.

Beyond multiomics, the modeling dimensions of PDOs can be further expanded when they are integrated with other experimental models and engineering platforms (Miserocchi et al., 2023; Feng et al., 2025). Within this framework, PDOs can serve as an intermediate layer connecting different scales of investigation rather than as stand-alone surrogates of tumors. Reductionist systems such as 2D cultures remain useful for rapid perturbation and candidate nomination, but their findings often need to be re-evaluated in more patient-proximal models. This need is underscored by Monberg et al., who showed that widely used pancreatic preclinical models can harbor occult genomic and transcriptomic polyclonality, display custodial variation across laboratories, and undergo substantial transcriptional divergence when shifted from 2D to 3D growth conditions, thereby highlighting the instability of low-context systems over time (Monberg et al., 2022). Thorel et al. similarly demonstrated, using ovarian clear cell carcinoma models derived from the same tumor, that PDTOs and the matched PDX, but not 2D cell lines, reproduced both histologic heterogeneity and the patient’s resistance to carboplatin, doxorubicin, and gemcitabine (Thorel et al., 2023). A comparable tiered strategy was reported by Soosainathan et al., who first identified CDK9-targeting vulnerability in 2D and 3D resistant ER-positive breast cancer models, then evaluated this dependency in PDOs derived from PDX tumors, and finally validated the therapeutic effect in resistant PDXs (Soosainathan et al., 2024). PDOs can also connect genetically defined systems to *in vivo* complexity. Taş et al. generated organoids from Kras-driven GEM lung adenocarcinomas, showed that they retained the genomic and histopathologic features of the parental tumors, enabled *ex vivo* drug-combination screening, and could subsequently be transplanted for *in vivo* validation (Taş et al., 2025). Likewise, Mucciolo et al. used PDAC organoid-derived cultures to identify EGFR/ERBB2-dependent myCAF states, but required orthotopic co-transplantation with fibroblasts to demonstrate their prometastatic function *in vivo* (Mucciolo et al., 2024). PDOs are most informative when positioned between fast reductionist models and higher-context *in vivo* systems, because they allow candidate mechanisms to be filtered in patient-specific three-dimensional settings before being further tested under stromal and organism-level constraints (Liao et al., 2025).

More complex coculture systems, engineering platforms, and selective *in vivo* models can further extend the modeling dimensions of PDOs by restoring microenvironmental and organism-level constraints that epithelial-only organoids do not fully capture. In colorectal cancer, Farin et al. established a matched PDO–CAF biobank and showed that stromal coculture or xenotransplantation restored transcriptomic fidelity and uncovered CMS4-specific resistance to SN-38 and gefitinib, thereby revealing subtype-specific resistance programs that were incompletely represented in standard PDOs (Farin et al., 2023). A related form of extension is illustrated by Schwörer et al. in pancreatic cancer, who combined PDAC organoid–stellate cell coculture with hypoxic conditioning and showed that hypoxia synergized with tumor cell-derived cytokines to drive an inflammatory CAF phenotype through HIF-1α-dependent signaling, indicating that PDO-based coculture can be used to model stromal state transitions imposed by local metabolic and cytokine gradients. (Schwörer et al., 2023). Engineering platforms provide a different form of extension. Han et al. developed bioprinted PDO arrays that preserved patient-specific morphology while recreating key extrinsic features of the tumor microenvironment, including elevated matrix stiffness and hypoxic conditions, thus enabling organoid phenotypes to be examined under more standardized yet biologically relevant constraints. Immune-related systems expand PDOs in yet another direction (Han et al., 2025).

No single preclinical model can recapitulate the full tumor ecosystem. Accordingly, these systems should not be viewed as competing substitutes, but rather as functionally complementary and hierarchically organized layers of evidence (Miserocchi et al., 2023).

## 7 Conclusion and discussions

From the perspective of tumor heterogeneity, organoids are not simply an *in vitro* model that is “more patient like.” Rather, they offer a balance between patient proximal fidelity, molecular resolution, and experimental controllability that is difficult to reproduce with other platforms (Thorel et al., 2023; Lencioni et al., 2024). A distinctive contribution of PDOs lies in redefining the operational unit of heterogeneity research. By retaining patient-specific genetic context while providing a standardized 3D experimental substrate, PDOs convert variation observed across tissues, regions, lesions, and time points into comparable *ex vivo* phenotypes and mechanistic tests. This, in turn, allows heterogeneity to be interrogated as measurable and perturbable state distributions, instead of being reduced to a single clone, a single lesion, or a single endpoint readout.

Within this framework, PDOs are best positioned as a hub in two complementary senses. First, they serve as a technical hub that organizes an expanding family of derivative systems. Immune retaining cultures, coculture assembloids, engineered matrices, vascularized organoids, organoid-on-a-chip platforms, and bioprinting approaches can be viewed as modular extensions built around a common PDO backbone, each aiming to restore specific missing heterogeneity dimensions while preserving patient specificity. Second, PDOs function as a translational hub that bridges *in vivo* and *in vitro* and links basic mechanism to clinical practice. Patient tissue and liquid biopsy define the *in vivo* heterogeneity landscape (Liu et al., 2023; Zhao et al., 2025), PDOs provide an *ex vivo* space where candidate programs can be perturbed with controllable readouts, and PDO derived xenografts or related *in vivo* models reintroduce systemic constraints (Merrill et al., 2024). In parallel, multicenter standardized PDO biobanks that integrate genomic and epigenomic profiling, spatial omics, and functional readouts such as drug response, immune activity, and evolutionary trajectories could enable a functional atlas of heterogeneity and support patient stratification defined by states and programs rather than by single genes, thereby informing clinical treatment selection and the design of rational combination strategies.

When guided by biological questions rather than by model centric comparisons, embedding PDOs within this chain supports a tiered validation path from molecular features to functional vulnerabilities to therapeutic design. Accordingly, a more promising direction for organoid based heterogeneity research is not to further strengthen any single model in isolation, but to use PDOs as a hub to reconfigure an across scale and across model workflow.

Overall, organoid technologies have not simplified the complexity of tumor heterogeneity; instead, they provide a new operational interface that makes this complexity observable, perturbable, and rewritable along controlled three dimensional spatial structures and experimental time axes. The central challenge is not to prove that PDOs are sufficiently patient like, but to define and respect their boundary conditions while embedding organoid platforms into the full translational chain from molecular stratification and heterogeneity deconstruction to therapeutic decision making and trial design, so that tumor heterogeneity becomes not merely a *post hoc* explanation for failure, but a starting point for prospective strategy development and the discovery of new vulnerabilities.
